# Topical application of Tea leaf-derived nanovesicles reduce melanogenesis by modulating the miR-828b/*MYB*4 axis: better permeability and therapeutic efficacy than conventional tea extracts

**DOI:** 10.1016/j.mtbio.2025.102108

**Published:** 2025-07-17

**Authors:** Fuyong Lin, Ting Wang, Jinwei Ai, Junxiang Wang, Chushan Huang, Wenrong Tian, Tianyang Lan, Lixia Fu, Xiaosong Chen

**Affiliations:** a Department of Plastic Surgery and Regenerative Medicine, Fujian Medical University Union Hospital, Fuzhou, 350001, China; b Department of Plastic Surgery and Regenerative Medicine Institute, Fujian Medical University, Fuzhou, 350001, China; c Engineering Research Center of Tissue and Organ Regeneration, Fujian Province University, 350001, China; d Department Three of Orthopedics/Plastic Surgery, Hubei University of Medicine, Xiangyang No.1 People's Hospital, Xiangyang, 441000, China; e College of Materials and Chemical Engineering, Minjiang University, Fuzhou, 350108, China

**Keywords:** Tea leaf-derived nanovesicles (TLNVs), miR-828b, *MYB*4, Skin pigmentation, Natural nanomaterials

## Abstract

Over-pigmentation of skin caused by excessive melanin production faces the challenges of limited therapeutic effects and safety, the conventional tea leaf extract (TET) for pigmentation treatment has the disadvantages of residual harmful substances, low penetration efficiency, here we propose the tea leaf-derived nanovesicles (TLNVs) as natural nanomaterials that combine bioactive components in tea leaves and exosome-like delivery advantages for targeting over-pigmentation treatment. This study extracted TLNVs from fresh tea leaves by ultracentrifugation and characterized them by transmission electron microscopy (TEM), nanoparticle tracking analysis (NTA) and secondary metabolites composition analysis. In vitro, TLNVs exhibited stronger radical scavenging ability and tyrosinase inhibitory effect than conventional tea leaf extract, while inhibiting B16-F10 cell proliferation and melanin synthesis. Based on these in vitro results, we further evaluated the anti-pigmentation effects of TLNVs in an uvB-induced pigmented mouse model, in which TLNVs markedly reduced epidermal melanin deposition and epidermal thickness while increasing dermal thickness and collagen volume fraction. TLNVs effectively suppressed the expression of inflammatory cytokines (TNF-α and IL-1β) and promoted melanoautophagy by upregulating LC3B and downregulating P62. Moreover, confocal laser scanning microscopy (CLSM) analysis of fluorescently labeled TLNVs confirmed their penetration ability into the deep dermis, reaching approximately 200 μm. Mechanistic studies demonstrated that miR-828b in TLNVs directly targeted *MYB*4 via PI3K/AKT pathway and downregulated melanogenesis regulators such as MITF and TYR. to reduce melanin production. Overexpression of *MYB*4 reversed the inhibitory effects of miR-828b on melanogenesis, confirming the specificity of this regulatory axis. This study is the first to confirm that TLNVs, as natural nanomaterials, exert multifunctional properties in combating skin pigmentation through the miR-828b/*MYB*4 axis. These include antioxidation, anti-inflammation, and the promotion of autophagy activity. TLNVs with high transdermal permeability and low toxicity provide a safer strategy for coping with pigmented skin diseases and sustainable tea leaf resource utilization.

## Highlights

• Topically applied Tea leaf-derived nanovesicles (TLNVs) effectively inhibit pigmentation and outperform conventional tea extracts.
• TLNVs deliver miR-828b to target *MYB*4 and downregulate melanin synthesis.
• TLNVs are rich in miRNA, polyphenols and flavonoids, and exhibit superior antioxidant, anti-inflammatory and pro-autophagy effects.
• TLNVs achieve deep dermal penetration (about 200 μm) with high transdermal efficiency.
• The efficacy of TLNVs in inhibiting pigmentation was confirmed using both classic melanogenesis cell models and pigmented mice models.

## Graphical abstract

**Figure:**
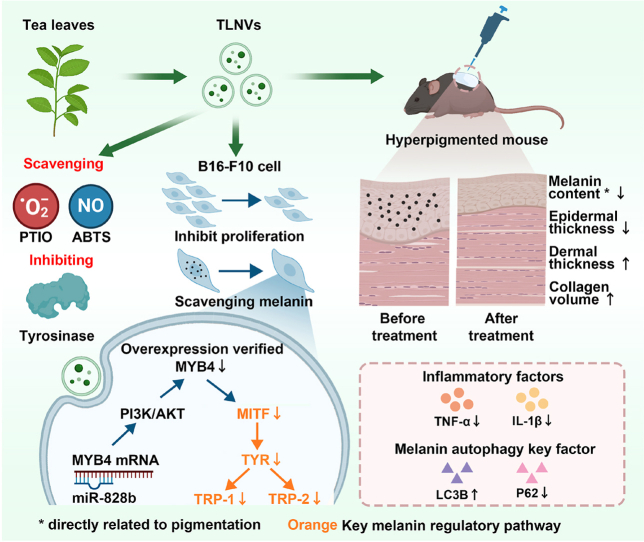

## 1 Introduction

Skin hyperpigmentation, a prevalent dermatological issue, is typically characterized by increased melanin production and is a distinguishing feature of various skin pathologies such as chloasma, freckles, and post-inflammatory hyperpigmentation [1,2]. This condition, while primarily affecting physical appearance, can also significantly impact mental health [3]. Melanin production is regulated by a myriad of internal and external factors including ultraviolet radiation, inflammation, hormonal fluctuations, genetic predispositions, and dietary habits [[4], [5], [6], [7]]. Current treatments for skin hyperpigmentation, which include drug therapy, physical therapy, and chemical peeling, often present risks for side effects like skin irritation, redness, and gastrointestinal discomfort [[8], [9], [10]]. In light of increasing public demand for safe and effective treatments for skin health and beauty, the exploration for natural substances that can effectively regulate melanin production without toxicity has emerged as an important research focus within the fields of dermatology and cosmetic medicine [11].

Tea leaf (*Camellia sinensis*) is a subject of extensive research and utilization in the realm of skin care, owing to its abundance of bioactive constituents such as polyphenols, flavonoids, and catechins [[12], [13], [14]]. The antioxidant and anti-inflammatory attributes of tea extracts (TET) are well-established, as evidenced by a myriad of studies [15,16]. These active components have been shown to diminish melanin formation through the scavenging of free radicals, the inhibition of inflammatory factor release, and the suppression of tyrosinase activity [17]. Mechanistically, melanin synthesis is regulated by key pathways such as the PI3K/AKT, MAPK, and Wnt/β-catenin signaling pathways, which govern the expression and activation of melanogenesis-associated genes including MITF, TYR, TRP-1, and TRP-2 [18,19]. Targeting these signaling cascades has become an important strategy for developing anti-hyperpigmentation agents. However, the practical utilization of TET encounters limitations that hinder their full effectiveness. These include a large molecular weight, poor permeability and stability, as well as residual harmful solvents from the extraction process [20].

In recent years, exosomes have emerged as a novel type of nano-scale bioactive carrier, garnering widespread attention due to their unique structure and functions [21]. Exosomes, characterized by their natural cellular membrane structure, are capable of carrying and delivering a variety of bioactive substances, including proteins, nucleic acids, and lipids. They demonstrate efficient intracellular transmission capabilities and excellent biocompatibility [[22], [23], [24], [25]]. Given their potential applications in drug delivery, gene therapy, and tissue repair, exosomes are viewed as a promising natural nanocarrier with broad prospects [[26], [27], [28], [29]]. Tea leaf-derived nanovesicles (TLNVs), a new type of natural nanomaterial, amalgamate the bioactive components of tea leaves with the delivery advantages of exosomes. They may offer significant benefits for skin whitening and anti-hyperpigmentation [30]. Preliminary studies indicate that TLNVs can effectively deliver the active components found in tea leaves, demonstrating high bioavailability and stability. However, their specific effects on reducing melanin production and improving skin hyperpigmentation have not been systematically studied and verified [[31], [32], [33]].

**Scheme 1:**
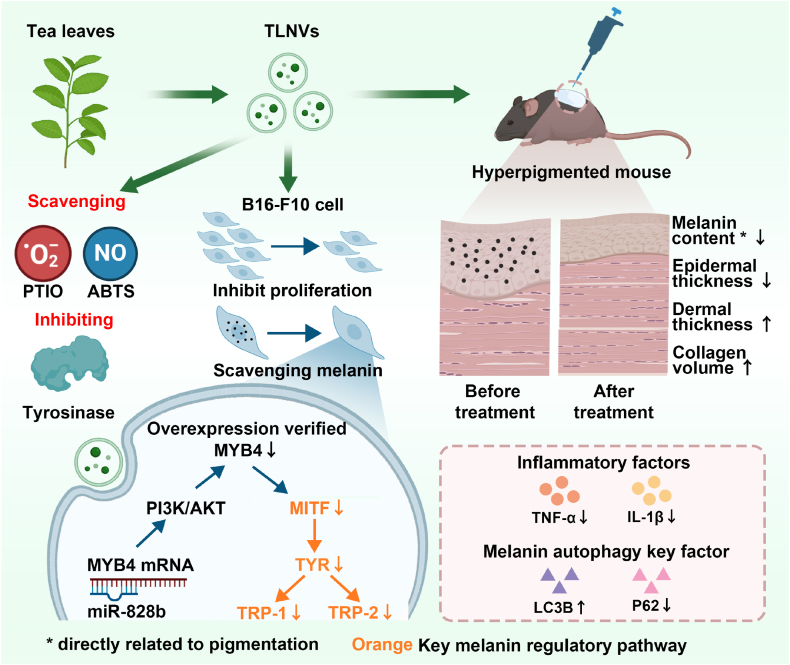
Schematic flowchart of the role and mechanism of Tea leaf-derived nanovesicles in improving pigmentation.

Tea leaves from various tea tree species may exhibit significant differences in their plant chemical composition, attributable to variations in the cultivation environment. These differences are particularly evident in the content of catechins, polyphenols, and flavonoids, which consequently influence their biological activities. In this study, we selected Fujian Fudingdabai tea leaves, a variety known for its high concentrations of polyphenols and catechins, to systematically evaluate the potential of TLNVs to enhance skin pigmentation. We then compared its efficacy with that of traditional tea leaf extract and the positive control, arbutin. Initially, TLNVs were successfully isolated and purified from tea leaves, then comprehensively characterized using transmission electron microscopy (TEM), nanoparticle tracking analysis (NTA), metabolomics, secondary metabolite composition analysis, and miRNA sequencing. Subsequently, we assessed the antioxidant capacity of TLNVs, their impact on the proliferation of B16-F10 mouse melanoma cells, and their potential to promote intracellular melanin clearance. Furthermore, animal experiments were conducted, with pathological staining, inflammation factor and key factors of melanin autophagy immunohistochemistry employed (Scheme 1).

## 2 Materials and methods

### 2.1 Isolation and characterization of TLNVs

Fresh tea leaves (*Camellia sinensis*, Fujian Dabai variety) are picked from the tea planting base of Fujian Agriculture and Forestry University in Fuzhou, Fujian Province, China. Upon harvesting the fresh leaves of the tea plant, a homogenate was created using a cell disruptor. This was followed by the addition of 1 % cellulase and 0.5 % pectinase for a 12-h digestion period. The subsequent centrifugation process was performed in a specific order: an initial centrifugation at 1000*g* for 20 min to eliminate the protoplasts, a secondary centrifugation at 5000*g* for 40 min to remove dead cells, and a final centrifugation at 10000*g* for 1 h to discard cell debris. The resulting supernatant was then filtered through a 0.22 μm membrane filter. This was followed by ultracentrifugation at 100000 g for 1 h to separate and purify, and the final product was resuspended in PBS to yield TLNVs. The protein concentration of TLNVs was measured using a BCA protein concentration assay kit (Beyotime Biotechnology, China). The morphology of these TLNVs was observed via transmission electron microscopy (Hitachi HT-7700). The particle size distribution and concentration were determined using a nanoparticle analyzer (NTA, Beckman Coulter, USA), while the zeta potential was detected with a nanoparticle tracking analyzer (PARTICLE METRIX, PMX120, USA). The miRNA sequencing of the TLNVs was conducted by Shanghai Aiputikang Biotechnology Co., Ltd., utilizing the TruSeq Small RNA Sample Prep Kit (Illumina, San Diego, USA) for the construction of the small RNA library. The sequencing platform used was the Illumina HiSeq 2000/2500. The secondary metabolite composition of the TLNVs was analyzed using ultra-high performance liquid chromatography–tandem mass spectrometry (LC–MS/MS).

### 2.2 PTIO radical scavenging activity

Prepare a PTIO working solution at a concentration of 0.2 mg/mL. Add 40 μL of TLNVs at concentrations of 10, 50, and 100 μg/mL to a 96-well plate, followed by 160 μL of the PTIO solution, and mix. Incubate the sample in a constant temperature incubator at 37 °C for 30 min. Measure the sample's absorbance value (A1) at a wavelength of 557 nm. Define the absorbance of the PTIO working solution mixed with PBS as (A0), and the absorbance of TLNVs mixed with PBS as (A2). Use TET (PY0812LC, Nanjing Puyi Biotechnology, CHINA) and arbutin as controls in the experiment. TET is a commercially available green tea extract used as a cosmetic ingredient. The main constituents are catechins (98.1 %), including epigallocatechin gallate (EGCG, 54.9 %) and gallate catechin (GCG, 2.2 %). It also contains small amounts of other components such as caffeine (0.5 %). Calculate the clearance rate using the formula: [1-(A1-A2)/A0] × 100 %.

### 2.3 ABTS radical scavenging activity

Prepare an ABTS solution with a concentration of 3.84 mg/mL and a potassium persulfate solution with a concentration of 0.67 mg/mL. After combining these solutions in equal volumes, the working ABTS solution is obtained. Prior to utilization, adjust the absorbance of this working ABTS solution to between 0.2 and 0.7 using distilled water. Add 50 μL of TLNVs at concentrations of 10 μg/mL, 50 μg/mL, and 100 μg/mL to separate wells in a 96-well plate, then mix with 150 μL of the ABTS working solution. Allow to react at room temperature for 10 min, then measure the sample's absorbance (B1) at a wavelength of 734 nm. The absorbance of the ABTS working solution mixed with PBS is denoted as (B0), while the absorbance of TLNVs mixed with PBS is denoted as (B2). Use TET and arbutin as controls in the experiment and calculate the clearance rate according to formula [1 - (B1 - B2)/B0] × 100 %.

### 2.4 Tyrosinase activity assay

The tyrosinase inhibitory activity of TLNVs (10, 50, 100 μg/mL) was measured using a BOXBIO tyrosinase activity assay kit (AKAM010M, Beijing Box Shenggong Technology, CHINA). The enzyme marker was preheated for 30 min, the wavelength was adjusted to 475 nm, and it was zeroed with distilled water. A volume of 20 μL TLNVs and 180 μL of Reagent One (dissolved in the extract solution provided in the kit) were sequentially added to a 96-well plate, mixed thoroughly immediately, and the timing was initiated. The absorbance at 475 nm at 10 s (total time) was measured as A1; after an accurate reaction for 180 s, the absorbance at 475 nm at 190 s (total time) was measured as A2, and ΔA = A2 - A1. The tyrosinase activity (U/mL) was calculated as 180.18 × ΔA, where ΔA is the change in absorbance at 475 nm per minute. The coefficient 180.18 is derived from the standard formula based on the molar extinction coefficient of dopachrome and assay conditions. The experiments were performed with TET and arbutin (A well-known tyrosinase inhibitor is often used as a positive control in melanin inhibition experiments) as controls, and the tyrosinase activity measured in the blank control (without any inhibitors or test samples) was recorded as B1, and the tyrosinase activity measured after adding the test sample was recorded as B2. The relative enzyme activity (%) was obtained from the following formula: B2/B1 × 100 %.

### 2.5 Cell culture

B16-F10 mouse melanoma cells were acquired from Wuhan Procell Life Science and Technology Co., Ltd. These cells were cultured in DMEM medium supplemented with 10 % fetal bovine serum and 1 % penicillin–streptomycin, maintained at 37 °C within an incubator offering a 5 % CO_2_ environment. Upon reaching approximately 80 % confluence, the cells were trypsinized using 0.25 % trypsin, subsequently passaged, and utilized in their logarithmic growth phase for experimentation.

### 2.6 Cell proliferation assay

Upon treatment of B16-F10 cells with 10, 50, and 100 μg/mL TLNVs for a duration of 48 h, an EdU working solution was introduced for labeling. Subsequent to the labeling process, cells were fixed using 4 % polyformaldehyde for a period of 15 min. This was followed by permeabilization treatment utilizing PBS containing 0.3 % Triton X-100 for an additional 15 min. A click reaction solution was then applied, and the cells were incubated at room temperature for 30 min in darkness. Hoechst 33342 was used to stain the nucleus for a duration of 10 min. Images were procured via fluorescence microscopy, and cell counting was executed for quantitative analysis. The experiment employed TET and arbutin as controls.

### 2.7 Intracellular melanin electron microscopy detection

B16-F10 cells were exposed to TLNVs for 48 h, after which they were fixed in a solution containing 200 mM cacodylate buffer, 8 % paraformaldehyde, and 20 % polymethylaldehyde. Subsequently, the cells underwent dehydration through an ethanol gradient. Sections were then prepared using a Leica EMUC7 ultramicrotome (Leica) and collected on 200-mesh copper grids. These sections were stained with 1 % uranyl acetate and lead citrate before being imaged with a JEOL JEM-1010 transmission electron microscope (JEOL). The experiment utilized TET and arbutin as controls.

### 2.8 Intracellular melanin quantification

After a 48-h treatment of B16-F10 cells with TLNVs, the cells were rinsed with PBS and subjected to digestion with 2.5 % trypsin. Subsequently, the cell precipitate was collected. A 99 % melanin standard was serially diluted to concentrations of 625, 312.5, 156.25, 78.125, 39.063, 19.531, and 9.766 μg/mL. Both the samples and standards were then added to a 250 μL solution of 1 % DMSO-NaOH and lysed in an 80 °C water bath for 1 h. The resultant lysate was transferred to a 96-well plate, and its optical density (OD) value was measured at 405 nm using a microplate reader (EnVision, PerkinElmer, USA). The experiment employed TET and arbutin as controls. The melanin content was calculated using a standard curve ranging from 0 to 100 μg/mL of synthetic melanin in NaOH.

### 2.9 Western blot

Upon treatment of B16-F10 cells with TLNVs, the cells were collected and lysed using a lysis buffer to extract the total proteins. The protein concentration was ascertained using the BCA method, following which the proteins were separated via SDS-PAGE. Subsequently, the SDS-PAGE separated proteins were transferred onto a PVDF membrane. This membrane was then blocked for 1 h at room temperature using 5 % skim milk. It was subsequently incubated with the target protein-specific primary antibodies (MITF, TYR, TRP-1, TRP-2) and the corresponding secondary antibody sequentially, adhering to the recommended dilution ratios. Finally, the protein signal was detected using an enhanced chemiluminescence (ECL) substrate and quantified with ImageJ image analysis software. The experiment incorporated TET and arbutin as controls.

### 2.10 Pigmentation model

This study was approved by the Fujian Medical University's Ethics Animal Committee (ethical approval number: IACUC FJMU 2024-Y-0122). Following a week of adaptive feeding, C57BL/6 mice aged 6–8 weeks were randomly divided into five groups: blank, PBS treatment, arbutin treatment, TET treatment, and TLNVs treatment, each consisting of six mice. The UV irradiation dose was tailored to the minimum erythema dose in mice. Except for the blank group, all other groups were subjected to a fixed UVB dose post back depilation, with 200 mj/cm2 per exposure every alternate day, totaling 15 exposures. After 29 days, the mice in each group that had previously been exposed to UVB were topically treated with arbutin, TET, or TLNVs (all at 100 μg/mL) on their skin of back, applied every alternate day, for seven total applications. The PBS treatment group received an equivalent dose of PBS.

### 2.11 Histological analysis

Post-drug treatment, dorsal skin tissues from mice were collected and fixed in 4 % paraformaldehyde for 24 h. Subsequently, these tissues underwent dehydration, paraffin embedding, and were sectioned into 4 μm slices. These sections were stained with hematoxylin and eosin (HE), Masson's trichrome, and melanin for histological assessment. Additionally, the expression levels of IL-1β and TNF-α in the skin tissues were evaluated using immunohistochemistry. To gauge the extent of melanosome autophagy in vivo, we examined the expressions of key autophagy factors, LC3B and P62. All sections were visualized under an optical microscope (Nikon ECLIPSE Ni-U, Nikon Corporation, Japan) and quantitatively analyzed using the ImageJ image analysis software.

### 2.12 Percutaneous penetration ability

In order to assess the skin penetration capability of TLNVs, we utilized the lipophilic fluorescent dye Dil as a label for the TLNVs. This assay was conducted independently of the aforementioned sample processing steps to ensure specificity and avoid potential interference.TLNVs were combined with Dil at a ratio of 1:200 and incubated at 37 °C for half an hour in darkness. Subsequently, unbound dyes were eliminated through ultracentrifugation, and the TLNVs were resuspended in PBS. The labeled TLNVs were then applied to the depilated region on the backs of C57BL/6 mice (100 μL, 100 μg/mL). After allowing 2 h for interaction, skin tissues were harvested and preserved in 4 % paraformaldehyde. These tissues were cryosectioned and the distribution and penetration depth of Dil-labeled TLNVs within the skin were examined using a laser scanning confocal microscope (Leica TCS SP8, Ernst Leitz GmbH, Germany). The penetration ability was quantitatively evaluated through Z-axis scanning.

### 2.13 Proteomics

Proteomic analysis of dorsal skin from PBS and TLNVs treated mice was conducted to investigate the mechanism underlying TLNVs’ effects on pigmentation. Given that prior experiments showed TLNVs had better efficacy than TET, only TLNVs were included for mechanistic exploration. The process involved the use of liquid chromatography-tandem mass spectrometry (LC–MS/MS) technology to perform proteolysis, peptide purification, and detection. Furthermore, protein identification and quantitative analysis were executed using database search software such as MaxQuant. Subsequent to these steps, Gene Ontology (GO) function annotation and Kyoto Encyclopedia of Genes and Genomes (KEGG) pathway enrichment analyses were performed on the identified differentially expressed proteins. This was done to investigate the potential regulatory mechanism of tea exosomes in enhancing pigmentation in mouse skin.

### 2.14 Quantitative real-time PCR

Total RNA was extracted utilizing an RNA isolation kit (Vazyme, China), after which its concentration and purity were gauged via a spectrophotometer (Thermo Fisher Scientific). The reverse transcription process was executed using 2 μg of total RNA as the initial template, with a cDNA first-strand synthesis kit from Thermo Fisher Scientific Inc. Subsequent real-time fluorescence quantitative PCR detection was performed in a standardized 25 μL reaction volume using the SYBR Green system. The sequence details of the specific amplification primers are provided in the Supplementary Information (Table S1).

### 2.15 Dual-luciferase reporter gene assay

The potential target genes of miR-828b were screened through the target gene prediction website, http://www.microrna.org and *MYB*4 was identified as its potential target gene. Based on this bioinformatic prediction result, we designed and synthesized a luciferase reporter plasmid containing the 3′-UTR sequence of *MYB*4 (Wuhan Genecreate Bioengineering, China). To validate the direct regulatory effect of miR-828b on *MYB*4, HEK 293 cells were seeded into 24-well plates and cultured for 24 h, then co-transfected with mimics 828b and the reporter plasmid respectively. After 48 h, the luciferase activity was detected using the dual-luciferase reporter assay kit(JKR23008, Wuhan Genecreate Bioengineering, China).

### 2.16 Overexpression of *MYB*4 in B16-F10 cells

After inoculating B16-F10 cells in six-well plates to 50–70 %, they were transfected with *MYB*4 overexpression plasmid (pCDNA3.1-*MYB*4) and empty vector control (pCDNA3.1, Hunan Fenghui Biotechnology, China) using Lipofectamine 3000 (Invitrogen, USA). Each well was added with 2.5 μg plasmid DNA, 5 μL Lipofectamine 3000 and 250 μL Opti-MEM (Thermo Fisher Scientific, USA), and incubated without serum for 15 min, then added dropwise into the cell culture medium. After transfection for 6 h, the medium was replaced with fresh complete medium and cultured for another 48 h. Subsequently, cells were collected for qPCR detection of *MYB*4 mRNA and protein expression, and functional experiments such as EdU cell proliferation assay and melanin content determination were carried out.

### 2.17 Data analysis

In this study, all statistical data were presented as means accompanied by standard deviation. The statistical analysis was conducted using GraphPad Prism 10 software. Significant differences were represented by the following symbols: ∗∗∗∗p < 0.0001, ∗∗∗p < 0.001, ∗∗p < 0.01, and ∗p < 0.05. Each experiment was replicated three times.

## 3 Results

### 3.1 Identification and characterization of TLNVs

**Fig. 1:**
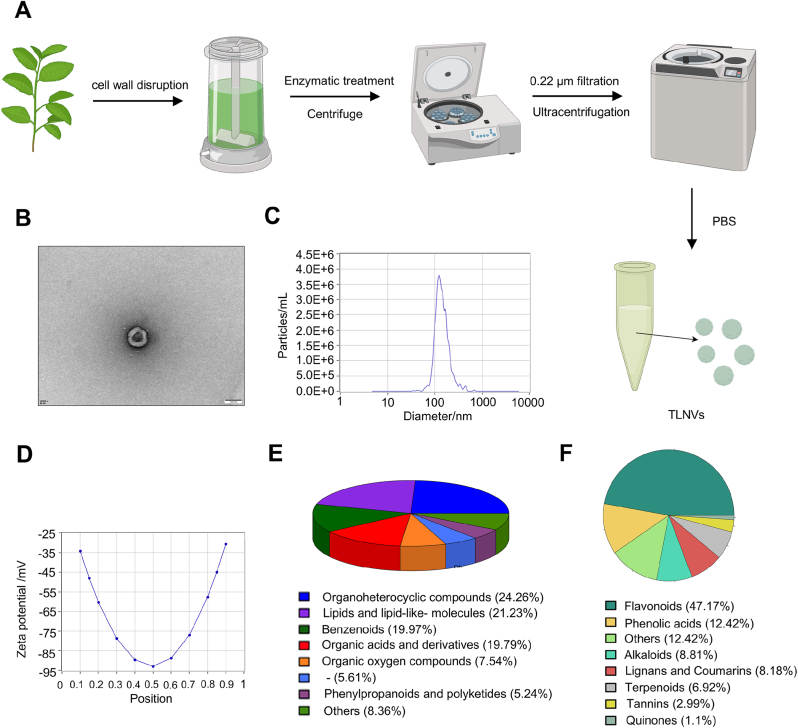
Isolation and characterization of TLNVs. **A**. The isolation process of TLNVs. **B**. Transmission electron microscopy observation of TLNVs (scale = 100 nm). **C**. Nanoparticle tracking analysis (NTA) of the TLNVs population showing a single peak pattern. **D**. Zeta potential analysis of TLNVs. **E**. Metabolomic analysis of TLNVs. **F**. Analysis of secondary metabolite composition of TLNVs.

The methodology for isolating TLNVs is depicted in Fig. 1A. In summary, recently harvested tea leaves were juiced and enzymatically digested. Subsequently, exosomes were isolated and purified via ultracentrifugation. Transmission electron microscopy (TEM) analysis revealed that the purified tea leaf exosomes exhibited a consistent morphology characterized by a distinct double membrane structure, consistent with the typical microscopic attributes of exosomes (Fig. 1B). Nanoparticle tracking analysis (NTA) demonstrated a relatively narrow particle size distribution for TLNVs, with an average diameter of 123.2 nm (Fig. 1C). The total yield of TLNVs extracted from 100 g of fresh tea leaves and resuspended in 2 mL of PBS was approximately 1.16 × 10^12^ particles, corresponding to 1.16 × 10^10^ particles per gram of fresh leaf. Based on BCA protein quantification, the total protein content of the TLNV suspension was 33.48 mg, resulting in a mass yield of 0.335 mg of TLNV protein per gram of fresh leaf. Zeta potential assessments revealed that the potential of TLNVs varied between −25 mV and −95 mV, with the most negative Zeta potential of approximately −90 mV observed at a position close to 0.5 (Fig. 1D). This suggests that TLNVs possess a strong negative charge and achieve optimal stability at 0.5. Such characteristics are likely to contribute to the excellent dispersibility and long-term stability of TLNVs, establishing a robust groundwork for their subsequent functionalities.

Metabolomics analysis revealed a diverse array of metabolites within TLNV bodies, underscoring the intricate nature of their exosomal components. The predominant metabolite categories comprised organoheterocyclic compounds (24.26 %), lipids and lipid-like molecules (21.23 %), and benzene ring type compounds (13.97 %). Notably, organic acids and their derivatives (13.79 %) and organooxygen compounds (7.54 %) were also significantly represented. Other notable metabolite categories encompassed phenylpropanoids and polyketides (5.24 %), organonitrogen compounds (2.57 %), organosulfur compounds (1.56 %), and nucleos, nucleotides, and analogues (2.45 %) (Fig. 1E). This metabolic profile highlights the potential of TLNVs for delivering biologically active substances and modulating cellular processes.

Tea leaves are rich in secondary metabolites, which contribute significantly to their biological activity. Hence, it is crucial to analyze the secondary metabolites present in TLNVs. The results reveal a diverse array of vital secondary metabolites in TLNVs. Among these, flavonoids constitute the highest proportion (47.17 %), followed by phenolic acids (12.42 %), tannins (2.99 %), alkaloids (8.81 %), lignans and coumarins (8.18 %), terpenoids (6.92 %), quinone compounds (1.10 %), and other secondary metabolites (Fig. 1F). In comparison to the singular components found in tea leaf extract, TLNVs contain not only active ingredients such as tea polyphenols but also substances with anti-inflammatory and antioxidant properties—including anthocyanins, tannins, phenolic amines, additional alkaloids, lignans, and coumarins. These components synergistically interact with each other through various mechanisms, potentially offering significant improvements to skin pigmentation.

miRNAs serve as key regulatory components within exosome-like nanovesicles, facilitating intercellular communication by modulating the expression of target genes. GO and KEGG enrichment analyses of miRNAs in TLNVs (Fig. S1) indicated that their predicted target genes are involved in transcription regulation, lipid metabolism, and hormone signal transduction. Among the enriched terms, “transcription factor activity,” “fatty acid biosynthesis,” and “plant hormone signal transduction” are particularly notable, as they correspond to biological processes that are commonly regulated by the PI3K/AKT signaling pathway. This correlation is further supported by previous studies demonstrating that bioactive compounds derived from tea can modulate PI3K/AKT signaling, thereby contributing to cellular homeostasis and pigmentation regulation.

### 3.2 TLNVs exhibit excellent free radical scavenging ability

**Fig. 2:**
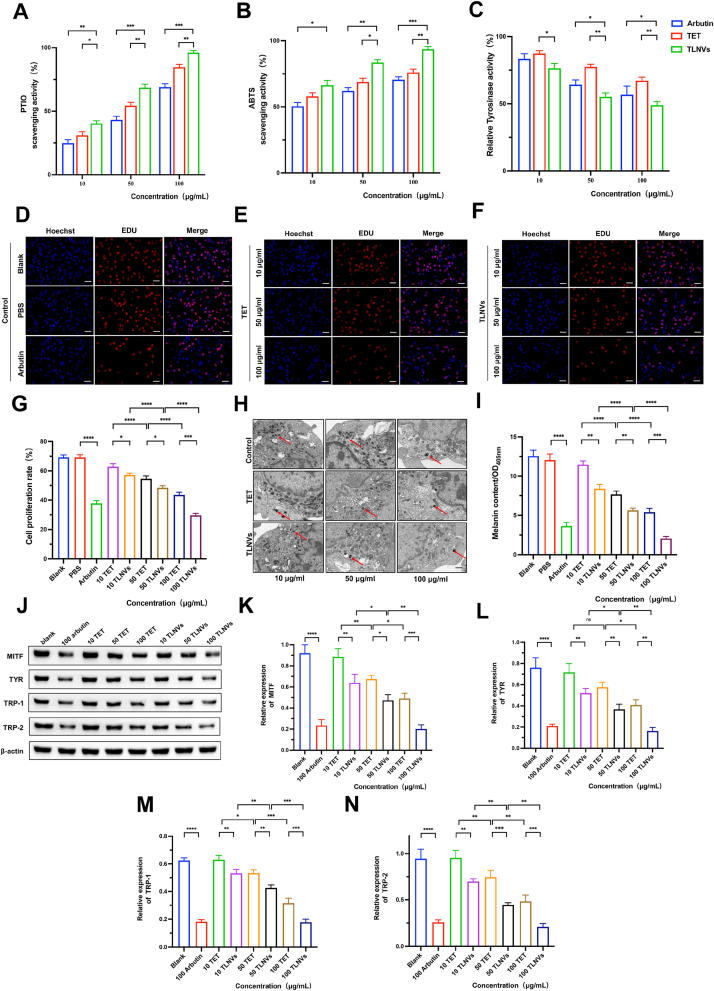
TLNVs can eliminate free radicals, inhibit tyrosinase activity and B16-F10 cell proliferation, and reduce melanin production and related protein expression. **A, B**. Scavenging activity of TLNVs on PTIO and ABTS free radicals (n = 5). **C**. Inhibition of tyrosinase activity by TLNVs (n = 5). **D, E, F**. Inhibition of B16-F10 cell proliferation by TLNVs (Scale bar = 100 μm). **G**. Quantitative analysis of B16-F10 cell proliferation (n = 5). **H**. Observation of reduced melanin production in B16-F10 cells under transmission electron microscope (Scale bar = 5 μm). **I**. Quantitative analysis of melanin production (n = 5). **J**. Expression of proteins related to melanin production. **K, L, M, N**. Quantitative analysis of protein expression of MITF, TYR, TRP-1 and TRP-2. ∗p < 0.05; ∗∗p < 0.01; ∗∗∗p < 0.001.

The antioxidant capacity of TLNVs and TET was assessed using the ABTS and PTIO radical scavenging activity assay, with arbutin serving as a control. Both TLNVs and TET exhibited significant radical scavenging capabilities (Fig. 2A and B). Notably, the scavenging activity of TLNVs escalated with increasing concentration. In comparison to TET, TLNVs demonstrated enhanced activity in scavenging ABTS and PTIO radicals. In both ABTS and PTIO radical scavenging assays, TLNVs demonstrated superior activity compared to TET and arbutin at all examined concentrations. The IC_50_ values for PTIO radical scavenging activity were 47.03 μg/mL for arbutin (R^2^ = 0.8997), 27.07 μg/mL for TET (R^2^ = 0.9133), and 16.61 μg/mL for TLNVs (R^2^ = 0.9525); for ABTS radical scavenging activity, the IC_50_ values were 10.08 μg/mL for arbutin (R^2^ = 0.9804), 3.66 μg/mL for TET (R^2^ = 0.9878), and 2.51 μg/mL for TLNVs (R^2^ = 0.9945). These findings suggest that TLNVs may possess superior antioxidant properties, and their efficacy is consistent across various radical scavenging assays.

### 3.3 TLNVs exhibited significant tyrosinase inhibitory effects

The inhibitory effects of TLNVs and TET on melanogenesis were evaluated by determining tyrosinase activity following treatment with TLNVs, TET, and the positive control, arbutin. Tyrosinase activity was significantly reduced in both the TLNVs and TET groups compared to the control group. Notably, TLNVs demonstrated an inhibitory effect comparable to that of arbutin, a known tyrosinase inhibitor, while TET moderately but significantly reduced enzyme activity (Fig. 2C). These findings suggest that TLNVs could effectively inhibit tyrosinase activity, thereby reducing melanin formation, indicating its potential application in the treatment of hyperpigmentation.

### 3.4 TLNVs markedly inhibited the proliferation of B16-F10 cells

In order to elucidate the regulatory impact of TLNVs on melanocyte proliferation, we administered varying concentrations (10, 50, and 100 μg/mL) of TLNVs to B16-F10 cells and evaluated their viability. The treatment with TLNVs resulted in a significant, dose-dependent inhibition of B16-F10 cell proliferation, with the most substantial inhibitory effect observed at the highest concentration (100 μg/mL). This trend was mirrored in the arbutin-treated group, thereby reinforcing its recognized role as a melanogenesis inhibitor. Conversely, TET treatment exhibited only a marginal decrease in cell proliferation relative to the control group (Fig. 2D–G). These results suggest that TLNVs possess the capacity to effectively inhibit the proliferation of B16-F10 cells, potentially contributing to their regulatory effect on melanocyte activity and thus ameliorating hyperpigmentation.

It should be noted that B16-F10 cells are a murine melanoma model commonly used for studying melanogenesis rather than normal skin physiology. Therefore, the observed inhibitory effect primarily reflects the impact of TLNVs on melanin-producing tumor cells. Although we did not evaluate the effects of TLNVs on normal skin cells in this study, such as keratinocytes or fibroblasts, further investigations are warranted to assess their biosafety and potential selectivity in future work.

### 3.5 TLNVs inhibit melanogenesis in B16-F10 cells

The impact of TLNVs on melanin regulation was assessed through a quantitative analysis of intracellular melanin content in B16-F10 cells following exposure to varying concentrations (10, 50, and 100 μg/mL) of TLNVs. The data revealed that TLNVs suppressed melanogenesis in a concentration-dependent manner, with peak efficacy observed at the highest concentration (100 μg/mL), as compared to untreated controls. The magnitude of the inhibitory effects exerted by TLNVs on melanin was comparable to that of the positive control, arbutin, demonstrating a corresponding dose response. Conversely, exposure to TET yielded statistically significant, albeit modest, anti-melanogenic effects within the examined concentration spectrum (Fig. 2H and I). These observations substantiate the conclusion that TLNVs serve as effective in vitro inhibitors of melanogenesis, highlighting their potential therapeutic utility in managing excessive pigmentation via the inhibition of melanin synthesis in B16-F10 cells.

### 3.6 TLNVs reduce the expression of pivotal proteins involved in melanogenesis

To thoroughly assess the regulatory impact of TLNVs on melanogenesis-associated proteins, B16-F10 cells were treated with concentrations of 10, 50, and 100 μg/mL TLNVs. The expression levels of MITF, TYR, TRP-1, and TRP-2 were subsequently measured. Our findings indicate that TLNVs effectively suppressed the expression of the melanogenesis-related proteins MITF, TYR, TRP-1, and TRP-2 at all tested concentrations, outperforming the positive control, arbutin, at certain concentrations (Fig. 2J–N). Importantly, TLNVs demonstrated distinct advantages over arbutin in decreasing the levels of melanogenesis-related proteins. While arbutin is renowned as a potent whitening agent, TLNVs, being natural active substances, not only offer comparable melanin inhibition but may also present broader biological activities and reduced cytotoxicity risks, suggesting their potential applications.

### 3.7 Animal experiments

**Fig. 3:**
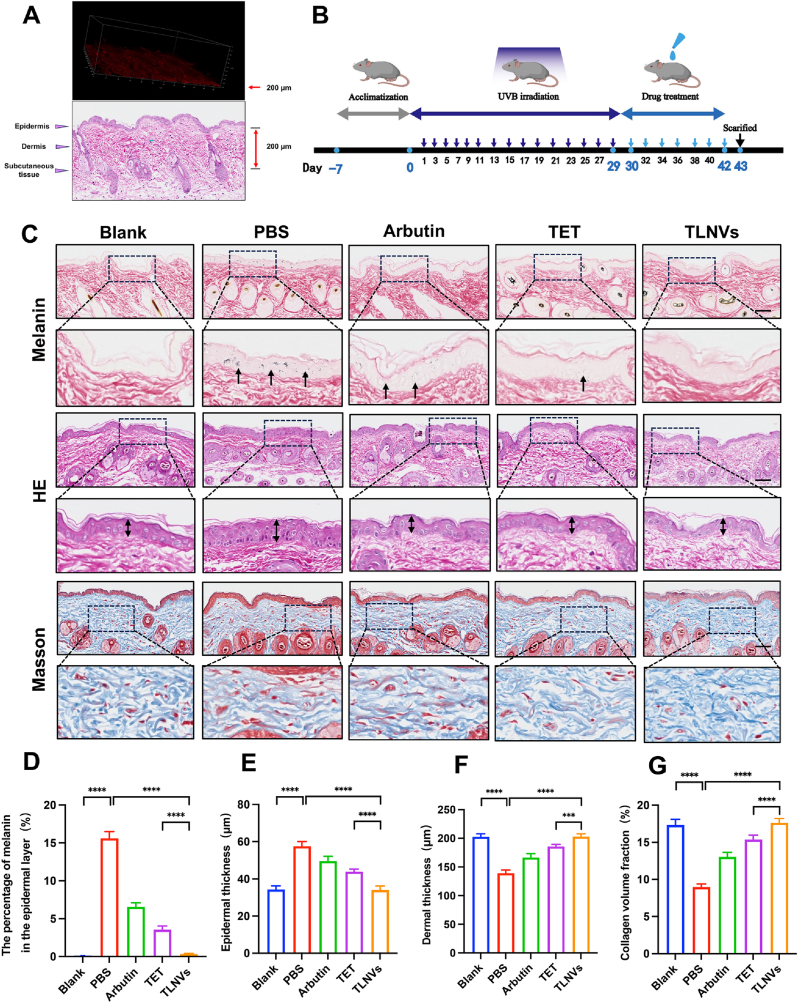
TLNVs improves skin pigmentation in mice. **A**. Flowchart of the construction of the pigmented animal model. **B**. Representative melanin staining (arrows point to melanin particles), HE staining (arrows indicate the thickness of the epidermal layer), and Masson staining (Scale bar = 100 μm) of mouse skin. **C**. Quantitative analysis of melanin content (n = 5). **D**. Quantitative analysis of epidermal layer thickness (n = 5). **E**. Quantitative analysis of dermal layer thickness (n = 5). **F**. Quantitative analysis of collagen volume fraction (n = 5). ∗p < 0.05; ∗∗p < 0.01; ∗∗∗p < 0.001; ∗∗∗∗p < 0.0001.

Through the use of laser scanning confocal microscopy, we observed that Dil-labeled TLNVs demonstrated significant penetration capabilities in the skin of C57BL/6 mice. The experimental data indicates that the penetration depth of these tea exosomal vesicles is approximately 200 μm. In conjunction with the Hematoxylin and Eosin staining results from the animal model, it was noted that the cumulative thickness of both the epidermis and dermis is also about 200 μm (Fig. 3A). This particular penetration depth is crucial for their role in melanin removal, considering that melanin is predominantly accumulated at the junction between the epidermis and dermis. These findings suggest that TLNVs possess the ability to permeate the stratum corneum, reach the deeper layers of the dermis, and exhibit efficient transdermal properties. Consequently, TLNVs hold the potential to significantly improve skin pigmentation through noninvasive applications, without the need for penetration-enhancing methods such as microneedles.

This study investigates the impact of TLNVs on skin pigmentation, utilizing a pigmented mouse model (Fig. 3B) with various control groups established, including a blank control (Blank), negative control (PBS), positive control (arbutin), and treatment groups for both TET and TLNVs. The melanin staining results reveal that, in comparison to the PBS group, the TLNVs treatment significantly curtails epidermal melanin deposition (Fig. 3C and D). Moreover, hematoxylin–eosin staining analysis indicates a substantial reduction in the thickness of the epidermis and an appreciable increase in the dermis thickness in the TLNVs group, suggesting the potential of TLNVs to enhance skin structure (Fig. 3C–E, F). Furthermore, Masson staining results demonstrate a significant elevation in the collagen volume fraction of the dermis following TLNVs treatment (Fig. 3C–G). Notably, TLNVs exhibited superior improvement effects compared to TET across multiple aspects. Through the reduction of melanin deposition, modulation of skin thickness, and stimulation of collagen generation, TLNVs exhibit promising application potential in ameliorating skin pigmentation and facilitating skin regeneration.

**Fig. 4:**
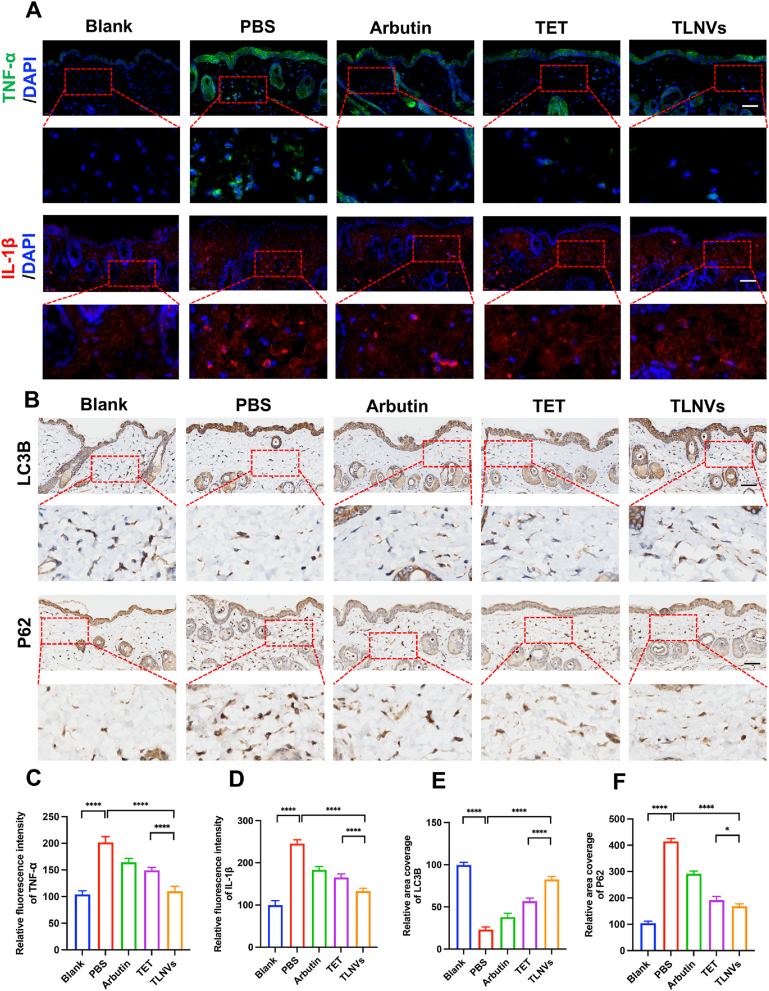
TLNVs can reduce inflammatory factors, increase the expression of LC3B and decrease the expression of key autophagy factor P62. **A**. Representative immunofluorescence staining of TNF-α and IL-1β in mouse skin (Scale bar = 100 μm). **B**. Representative immunofluorescence staining of LC3B and P62 in mouse skin (Scale bar = 100 μm). **C**. Quantitative analysis of TNF-α (n = 5). **D**. Quantitative analysis of IL-1β (n = 5). **E**. Quantitative analysis of LC3B (n = 5). **F**. Quantitative analysis of P62 (n = 5). ∗p < 0.05; ∗∗p < 0.01; ∗∗∗p < 0.001; ∗∗∗∗p < 0.0001.

Inflammatory responses and melanosome autophagy are pivotal in regulating skin pigmentation, and their dysregulation can precipitate pigment disorders. This study probed the impact of inflammatory reactions and melanosome autophagy on skin pigmentation by assessing the expression levels of inflammatory markers TNF-α and IL-1β, as well as key regulators of melanosome autophagy, namely LC3B and P62. Immunofluorescent staining revealed that, relative to the PBS control group, the TLNVs-treated group exhibited a significant diminution in the expression levels of TNF-α and IL-1β. Notably, their inhibitory effect surpassed that of arbutin and TET (Fig. 4A–C, D), indicating a pronounced anti-inflammatory efficacy of TLNVs. Moreover, post-TLNVs treatment, there was a marked augmentation in the expression of the melanosome autophagy-associated protein LC3B, concomitant with a notable decrease in P62 levels (Fig. 4B–E, F). This suggests that TLNVs might modulate melanin metabolism via the enhancement of the autophagy pathway. In essence, TLNVs mitigate the expression of pro-inflammatory cytokines TNF-α and IL-1β, ameliorate the inflammatory milieu, and foster melanin degradation through an autophagy pathway governed by LC3B and P62, underscoring its prospective utility in ameliorating skin pigmentation disorders.

### 3.8 Skin proteomic profiling of mice before and after dosing

**Fig. 5:**
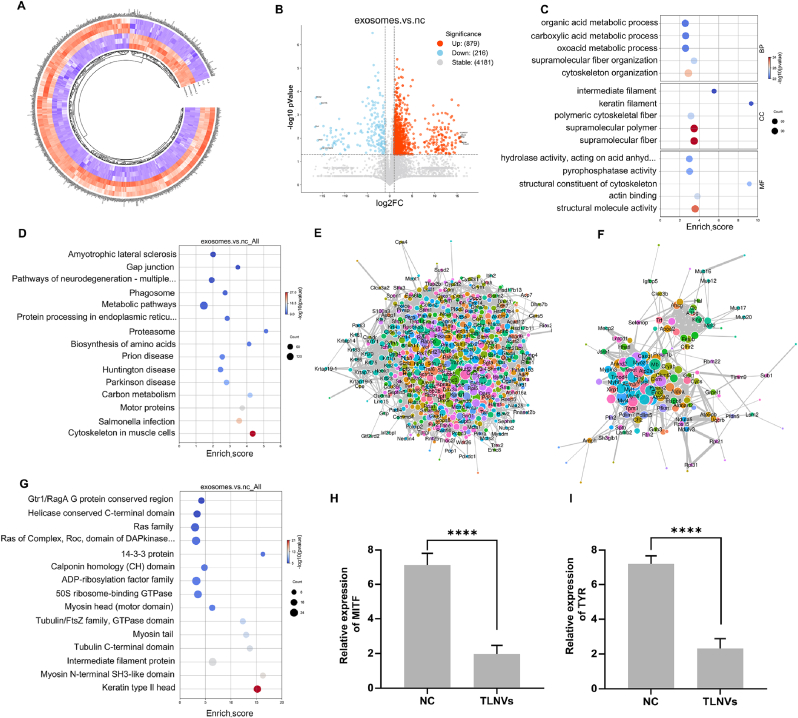
Regulation of melanogenesis-related protein expression after transdermal administration of TLNVs. **A**. Heatmap of differential clustering before and after TLNVs administration (n = 3). **B**. Volcano plot analysis before and after TLNVs administration. **C**. GO enrichment analysis of differential proteins. **D**. KEGG enrichment analysis of differential proteins. **E**. Protein interaction analysis of upregulated differential molecules. **F**. Protein interaction analysis of downregulated differential molecules. **G**. Domain enrichment analysis of differential proteins. **H**. Fluorescence quantitative expression analysis of MITF. **I**. Fluorescence quantitative expression analysis of TYR. ∗∗∗∗p < 0.0001.

In order to elucidate the role of TLNVs in skin pigmentation, we conducted a proteomic analysis on the skin tissues of the mice post-treatment. Initially, the differential protein expression was depicted through a heatmap, showcasing distinct disparities in protein expression between the experimental and control groups (Fig. 5A). Subsequent volcano plot analysis identified 879 up-regulated and 918 down-regulated proteins. Notably, BC048507 (a mitochondrial assembly protein), Dynll2 (dynein light chain involved in intracellular transport), Pomp (proteasome maturation protein), Apmap (adipocyte plasma membrane-associated protein linked to lipid metabolism), and Snap23 (a SNARE protein involved in vesicle trafficking) exhibited significant up-regulation, while Sh3bgr (associated with actin cytoskeleton remodeling), Atxn7l3b (linked to transcriptional regulation), Myot (a cytoskeletal protein related to structural integrity), Lmcd1 (a LIM domain protein involved in cardiac and muscle development), and LOC101056474 (uncharacterized) demonstrated pronounced down-regulation (Fig. 5B). These findings imply that TLNVs potentially influence the expression of a multitude of proteins.

To elucidate the biological roles of the differentially expressed proteins identified in our study, we performed GO functional annotation analysis. This revealed that the differentially expressed proteins identified through proteomic comparison between TLNV-treated and PBS-treated hyperpigmented mice were predominantly associated with biological processes such as organic acid metabolic processes, keratinocyte differentiation, and cytoskeleton organization, which have been previously reported to be involved in the regulation of skin pigmentation, suggesting that TLNVs might influence pigmentation by modulating cellular metabolism and cytoskeletal remodeling (Fig. 5C). KEGG pathway enrichment analysis indicated that these differential proteins were notably involved in critical signaling pathways such as amino acid biosynthesis, neurotransmitter pathway, and melanin metabolism, all of which may be intrinsically linked to the regulation of skin pigmentation (Fig. 5D). Furthermore, protein-protein interaction (PPI) network analysis demonstrated that the significantly upregulated proteins constituted an intricate interaction network (Fig. 5E), suggesting that some of these proteins could be pivotal in the pigmentation process. Conversely, the interaction analysis of the downregulated proteins highlighted significant alterations in several proteins associated with cell signal transduction (Fig. 5F), reinforcing the prospective role of tea plant exosomes in modulating skin pigmentation. Protein domain enrichment analysis revealed a marked enrichment of signaling regulatory domains like Gtr1/RagA G protein, Ras family, and 14-3-3 protein in the TLNVs treatment group (Fig. 5G). Additionally, the prominence of cytoskeleton-associated domains such as myosin head, intermediate filament protein, and keratin type II head underscores the potential impact of exosomes on cytoskeletal dynamics. Collectively, our findings propose that TLNVs might influence the pigmentation process by modulating signal transduction and cytoskeletal remodeling.

Based on the proteomics results, we found that the key regulators of melanogenesis MITF and TYR were significantly down-regulated, so we selected the two key melanogenesis-related genes MITF and TYR for further verification at the mRNA level. qPCR was performed using RNA samples from the PBS-treated group and the TLNVs-treated group to confirm whether the observed protein-level changes were also reflected in gene expression. This design allowed us to focus specifically on the regulatory effect of TLNVs. MITF and TYR mRNA levels were significantly reduced following TLNVs treatment, consistent with the proteomic data(Fig. 5H and I). Thus, TLNVs play a crucial role in ameliorating skin pigmentation by modulating key proteins such as MITF and TYR and regulating signaling pathways including PI3K/AKT and cytoskeleton-associated cascades, which are directly involved in melanogenesis and dermal remodeling.

### 3.9 MiR-828b regulates proliferation and melanogenesis in B16-F10 cells

**Fig. 6:**
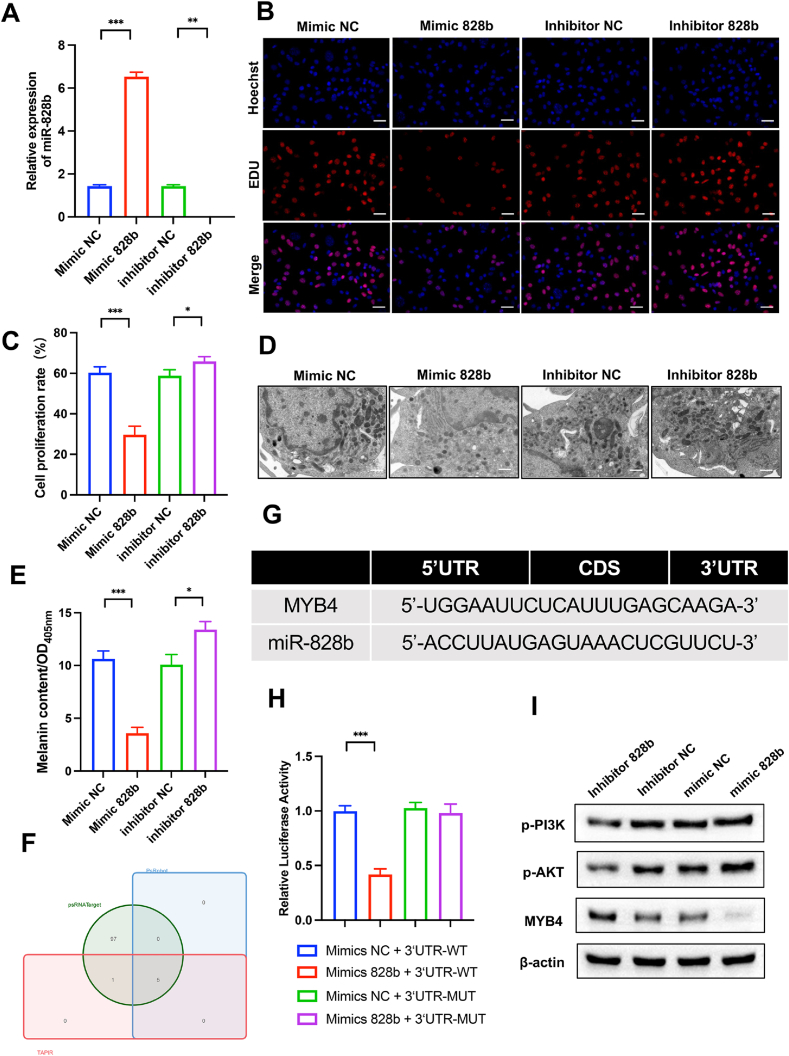
TLNVs possess good transdermal permeability and miR-828b can target MYB4 through the PI3K/AKT pathway to inhibit melanogenesis. **A**. TLNVs can penetrate to a depth of approximately 200 μm in the dermis of the skin. **B**. The target genes of miR-828b were predicted using a target gene prediction website. **C**. Schematic diagram of the putative binding site of miR-828b in the 3′-UTR of MYB4. **D**. EDU proliferation assay of the four groups of B16-F10 cells (Scale bar = 100 μm). **E**. Intracellular melanin in the four groups of B16-F10 cells was observed by transmission electron microscopy (Scale bar = 5 μm). **F**. Quantitative analysis of EDU cell proliferation. **G**. Quantitative analysis of intracellular melanin content. **H**. mRNA expression levels of miR-828b in B16-F10 cells treated with miR-828b mimics/inhibitors. **I**. Western blot detection of the expression of p-PI3K, p-AKT, and MYB4 in each group. **J**. Luciferase reporter gene assay confirmed that MYB4 is a target of miR-828b (n = 3). ∗p < 0.05; ∗∗p < 0.01; ∗∗∗p < 0.001. All relevant treatment groups were compared statistically, and only significant differences were highlighted in the figures to enhance readability. No significant difference was added for comparison.

Because miR-828b was found to be highly expressed in tea leaf-derived exosomes based on small RNA sequencing, and bioinformatic analysis predicted that miR-828b targets the transcription factor *MYB*4, which regulates pigmentation-related genes via pathways such as PI3K/AKT, and given that members of the miR-828 family have been reported to influence pigmentation-related biosynthesis in plants, we selected miR-828b as the focus of this study. In order to ascertain the biological function of miR-828b, we transfected miR-828b mimics into B16-F10 cells (Fig. 6A). The results from the EDU assay revealed that the miR-828b mimics significantly curtailed the proliferation of B16-F10 cells, whereas the miR-828b inhibitor enhanced cell proliferation (Fig. 6B and C). These findings suggest that miR-828b plays a pivotal role in regulating cell proliferation and indirectly demonstrates its capacity to inhibit melanogenesis. Moreover, through quantitative analysis of the melanin content in the cells, we further confirmed that miR-828b significantly inhibits the melaninogenesis of B16-F10 cells (Fig. 6D and E).

### 3.10 MiR-828b targets *MYB*4 by activating the PI3K/AKT pathway

To investigate the downstream regulatory mechanism of miR-828b, we initially predicted its potential target genes utilizing psRNA Target, PsRobot, and TAPIR algorithms, identifying 5 candidate targets (Fig. 6F). Subsequent analysis employing microRNA (http://www.microrna.org/) and TargetScan (http://www.targetscan.org/) databases pinpointed *MYB*4 as a prospective target of miR-828b (Fig. 6G). To further ascertain whether *MYB*4 is a direct target gene of miR-828b, luciferase reporter vectors (*MYB*4-WT and pmirGLO-3′UTR of *MYB*4-WT) were constructed and transfected into HEK 293 cells. The results indicated that overexpression of miR-828b significantly inhibited the luciferase activity in the *MYB*4-WT group (Fig. 6H). Furthermore, to confirm whether miR-828b regulates *MYB*4 via the PI3K/AKT pathway, B16-F10 cells were transfected with miR-828b mimic. The findings revealed that miR-828b reduced the protein expression level of *MYB*4 in cells and activated the PI3K/AKT pathway (Fig. 6I–S2), concurrently decreasing the expression levels of melanin-related regulatory factors MITF, TYR, TRP-1, and TRP-2 mRNA (Fig. S3). These results suggest that miR-828b inhibits *MYB*4 expression by binding to the conserved complementary sequence of *MYB*4 mRNA 3′UTR, thereby playing a regulatory role.

### 3.11 MiR-828b/*MYB*4 axis regulated B16-F10 cell function

**Fig. 7:**
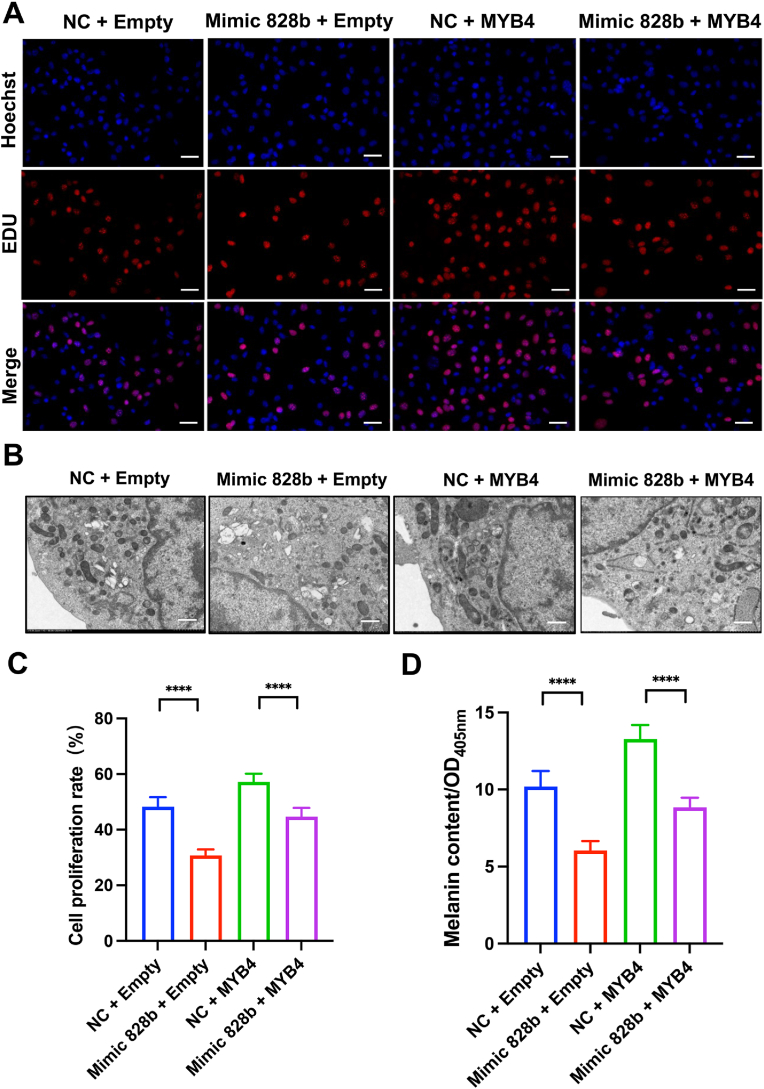
The miR-828b/*MYB*4 axis regulates the proliferation and melanogenesis of B16-F10 cells. **A**. EDU proliferation assay of B16-F10 cells in each group (Scale bar = 100 μm). **B**. Transmission electron microscopy observation of intracellular melanin in B16-F10 cells of each group (Scale bar = 5 μm). **C**. Quantitative analysis of EDU cell proliferation. **D**. Quantitative analysis of intracellular melanin content. ∗p < 0.05; ∗∗p < 0.01; ∗∗∗p < 0.001; ∗∗∗∗p < 0.0001. All relevant treatment groups were compared statistically, and only significant differences were highlighted in the figures to enhance readability. No significant difference was added for comparison.

In order to elucidate the biological function of the miR-828b/*MYB*4 axis, we overexpressed *MYB*4 in B16-F10 cells and verified its expression by measuring *MYB*4 mRNA levels (Fig. S4). Subsequently, we assessed the cell proliferation capacity of B16-F10 cells using EdU assays. Our findings revealed that *MYB*4 overexpression enhanced cell proliferation of B16-F10 cells and effectively counteracted the inhibitory effect of miR-828b mimics (Fig. 7A–C). Similarly, in the quantitative evaluation of melanin content, we noted a consistent pattern with that of cell proliferation. Specifically, *MYB*4 overexpression significantly boosted melanin synthesis by B16-F10 cells (Fig. 7B–D). In summary, our research illustrated that miR-828b inhibited both cell proliferation and melanogenesis of B16-F10 cells by targeting *MYB*4.

## 4 Discussion

Melanin plays a pivotal role in maintaining skin color and offering protection against UV damage. Ultraviolet irradiation damage leads to excessive keratinization and thickening of the stratum corneum, which destroys the normal barrier of the skin and the normal physiological functions of the skin, thus leading to excessive deposition of melanin. Nevertheless, irregularities in its metabolism frequently result in pigmentation disorders such as chloasma, post-inflammatory hyperpigmentation, and age spots [[34], [35]]. Hyperpigmentation not only impacts aesthetic beauty but also imposes a psychological burden on patients, particularly within the Oriental and dark-skinned populations [[36], [37]]. As society's emphasis on skin health and beauty grows, the question of how to safely and effectively ameliorate hyperpigmentation has emerged as a key research area in dermatology and aesthetics.

Current treatments for hyperpigmentation encompass chemical peeling, laser therapy, topical depigmenting agents (e.g., hydroquinone, azelaic acid, arbutin), and oral antioxidants [38,39]. However, these approaches often exhibit high side effects, inconsistent efficacy, and substantial recurrence rates [40]. In line with the rising emphasis on green therapy, natural plant-derived products have garnered considerable research attention [41]. Notably, tea leaves, abundant in active constituents such as catechins, flavonoids, alkaloids, and L-theanine, have demonstrated notable effects in antioxidation, anti-inflammation, and anti-photoaging [42]. Existing research indicates that tea leaf extracts can mitigate melanin synthesis by inhibiting tyrosinase activity, thus ameliorating hyperpigmentation [43]. Nonetheless, traditional tea leaf extracts possess certain drawbacks, including substantial molecular weight, limited stability, inadequate skin penetration, and potential residual harmful solvents from the extraction process, which collectively restrict their application in treating hyperpigmentation disorders [44].

This study pioneers the application of Tea Leaf Nano-vesicles (TLNVs) for enhancing skin pigmentation. TLNVs, owing to their natural origin, high safety profile, diminutive size, lipophilicity, and excellent transmembrane permeability, can more effectively penetrate the skin barrier to deliver active ingredients to melanocytes, thereby achieving precise treatment [45]. Furthermore, in contrast to traditional tea leaf extracts, exosomes which serve as carriers of intercellular information transmission not only contain a rich array of miRNA, proteins, and lipids but also demonstrate superior biocompatibility and targeting properties, thereby exhibiting enhanced efficacy in treating skin pigmentation disorders [46]. The findings of this study reveal that TLNVs exert a substantial inhibitory effect on melanin in both cell and animal models, underscoring their potential in treating hyperpigmentation. Taken together, the strong antioxidant capacity of TLNVs may reduce oxidative stress-mediated melanogenesis, while their excellent transdermal delivery ability ensures effective targeting of melanocytes. Moreover, the miRNA-mediated regulation of melanogenic pathways complements these physicochemical advantages, suggesting a multifactorial mechanism through which TLNVs exert their anti-pigmentation effects.

The biological impact of TLNVs is predominantly contingent upon the miRNA they enrich. In this investigation, employing bioinformatics predictions and dual-luciferase validation, we ascertained that miR-828b can directly target and inhibit *MYB*4 expression. Notably, *MYB*4 is a documented transcription factor instrumental in modulating plant phenylpropanoid metabolism and flavonoid synthesis [47]. We introduce, for the first time, the proposition that miR-828b down-regulates *MYB*4, thereby affecting the expression of tyrosinase, which is a central enzyme in the melanin synthesis pathway, ultimately reducing pigmentation. This groundbreaking discovery not only illuminates the potential of plant-derived exosomal miRNA to exert regulation across mammalian cells but also furnishes a novel perspective for comprehending the cross-species information transfer mechanism between plants and animals.

While this study has substantiated the role of TLNVs in suppressing melaninogenesis and delineated the molecular mechanisms involved, certain limitations persist. Notably, the experiments were predominantly conducted on the B16-F10 cell model and animal models; further clinical investigations are imperative to ascertain their efficacy and safety. Additionally, given the intricate composition of TLNVs, only the function of miR-828b has been elucidated thus far. The potential synergistic effects of other miRNAs or secondary metabolites warrant further exploration. Moreover, challenges remain in the large-scale extraction and purification of TLNVs, along with quality control measures. Future endeavors could focus on optimizing the extraction process and developing stable, efficient exosomal formulations.

## 5 Conclusions

Pigmented skin disorders and the limitations of conventional therapies highlight the need for safe, effective alternatives. This study demonstrates for the first time that TLNVs alleviate hyperpigmentation by targeting the miR-828b/*MYB*4 axis. TLNVs possess excellent transdermal permeability (approximately 200 μm), minimal cytotoxicity, and stable physicochemical properties, enabling efficient delivery of bioactive components. They exhibit strong antioxidant and tyrosinase-inhibiting activities and dose-dependently downregulate melanogenesis-related proteins, including MITF, TYR, TRP-1, and TRP-2. In vivo, TLNVs significantly reduce epidermal melanin accumulation, suppress the expression of inflammatory cytokines such as TNF-α and IL-1β, and promote melanoautophagy by increasing LC3B expression and decreasing P62 levels. Mechanistically, miR-828b in TLNVs inhibits *MYB*4 through the PI3K/AKT pathway, thereby modulating the expression of downstream pigmentation-related genes. These findings highlight the cross-kingdom regulatory potential of plant-derived nanovesicles and support the application of TLNVs as a promising natural, safe, and effective strategy for treating hyperpigmented skin disorders while promoting the high-value utilization of tea leaf resources.

## CRediT authorship contribution statement

**Fuyong Lin:** Writing – review & editing, Writing – original draft, Data curation, Conceptualization. **Ting Wang:** Formal analysis, Data curation. **Jinwei Ai:** Methodology, Investigation. **Junxiang Wang:** Software, Resources. **Chushan Huang:** Validation, Supervision. **Wenrong Tian:** Validation, Supervision. **Tianyang Lan:** Visualization. **Lixia Fu:** Visualization, Validation. **Xiaosong Chen:** Project administration, Funding acquisition.

## Declaration of competing interest

The authors declare no conflict of interest.

## Data Availability

Data will be made available on request.
