# Supplementary material for: Topical application of Tea leaf-derived nanovesicles reduce melanogenesis by modulating the miR-828b/MYB4 axis: better permeability and therapeutic efficacy than conventional tea extracts
Source: Mater Today Bio. 2025 Jul 17;34:102108. doi: 10.1016/j.mtbio.2025.102108 (PMC12305179; doi:10.1016/j.mtbio.2025.102108)
Supplement: Multimedia component 1 [file mmc1.docx]

# Supplementary Material

**Tea leaf-derived nanovesicles reduce melanogenesis by modulating the miR-828b/*MYB*4 axis: better permeability and therapeutic efficacy than conventional tea extracts**

Fuyong Lin^a,b,c,1^, Ting Wang^a,b,c,1^, Jinwei Ai^a,b,c,d,1^, Junxiang Wang^e^, Chushan Huang^a,b,c^, Wenrong Tian^a,b,c^, Tianyang Lan^a,b,c^, Lixia Fu^a,b,c^, Xiaosong Chen^a,b,c,*^

^a^Department of Plastic Surgery and Regenerative Medicine, Fujian Medical University Union Hospital, Fuzhou 350001, China.

^b^Department of Plastic Surgery and Regenerative Medicine Institute, Fujian Medical University, Fuzhou 350001, China.

^c^Engineering Research Center of Tissue and Organ Regeneration, Fujian Province University, 350001, China.

^d^Department Three of Orthopedics/Plastic Surgery, Xiangyang No.1 People’s Hospital, Hubei University Of Medicine, Xiangyang 441000, Hubei Province, China.

^e^College of Materials and Chemical Engineering, Minjiang University, Fuzhou, 350108, China.

^1^, These authors contributed equally to this work.

*****Corresponding authors at:** Department of Plastic Surgery and Regenerative Medicine, Fujian Medical University Union Hospital, Fuzhou 350001, China.

**E-mail addresses:** chenxiaosong74@163.com (X.Chen).

**Table S1. The qPCR primers used in the research.**

| **Gene** | **Sequence** |
| --- | --- |
| MITF | F：GATACCTTGTTTATAGTACCTTCT |
|  | R：TGAGATGGAGGGTTCAGAGAGG |
| TYR | F：CCTGGGCTATGGTGACTGTG |
|  | R：TGCTTCAGGGTCTGGTAGGT |
| TRP-1 | F：CCAGCCTACCTGGACCTCTT |
|  | R：GGGTCAGGTCCAGGTAGGTG |
| TRP-2 | F：GCTGGGACCTACAGCAGACA |
|  | R：CAGGTGCTGGTGTTGAGATG |
| miR-828b | F：ACACTCCAGCTGGGACCTTA |
|  | R：GTGCAGGGTCCGAGGT |
| MYB4 | F：TGGAATTCTCATTTGAGCAA |
|  | R：TCTTGCTCAAATGAGAATTCCA |

**A**

**
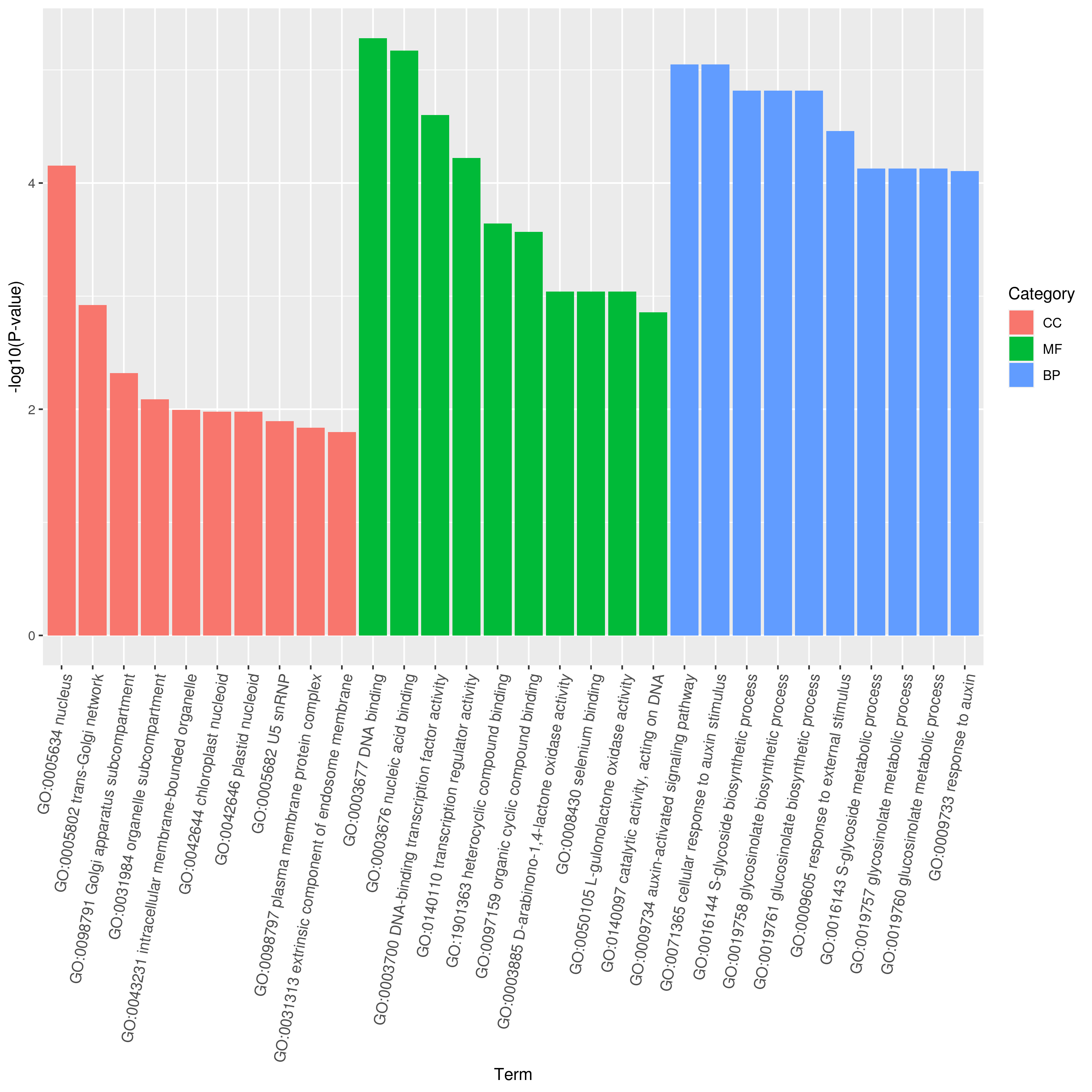
**

**B**

**
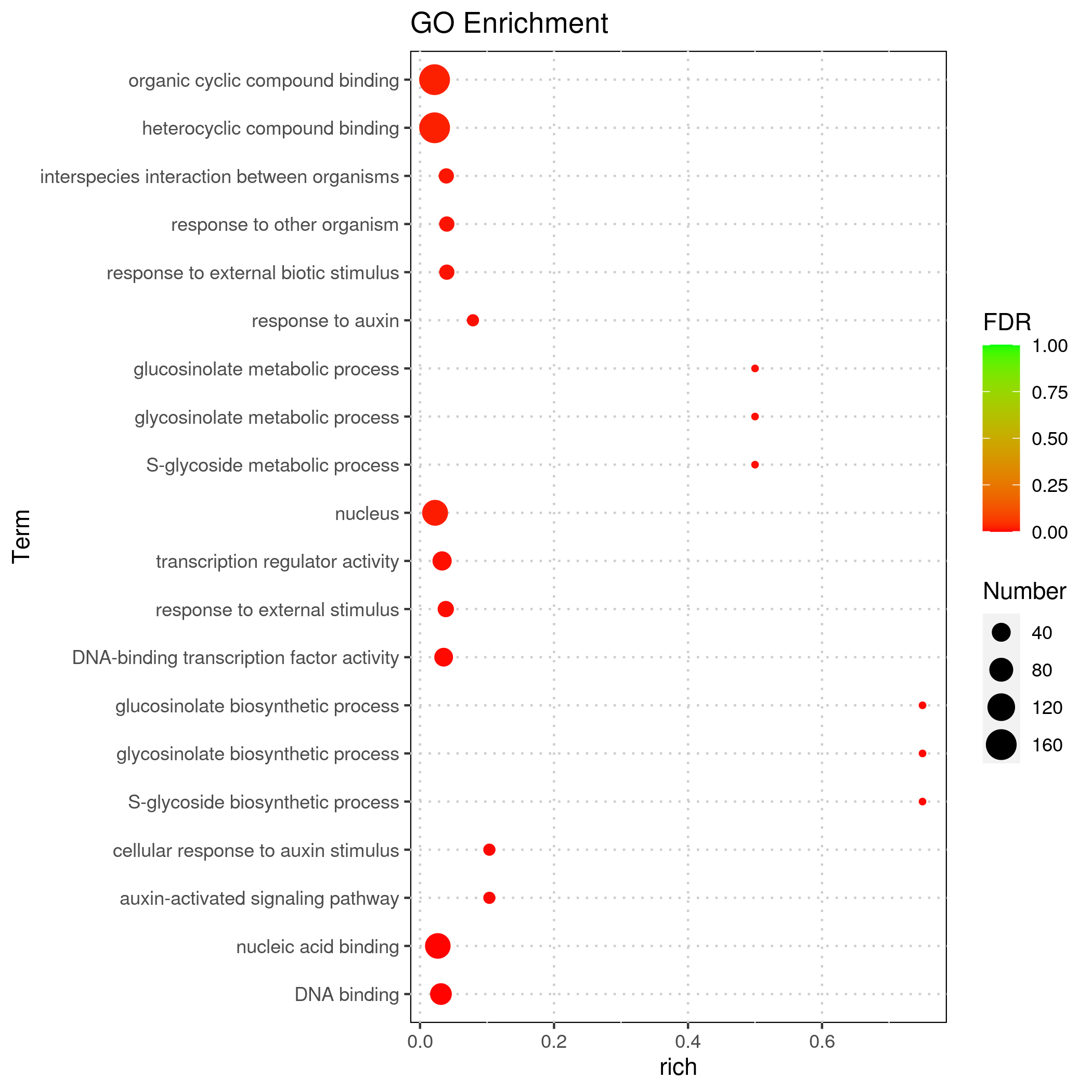
**

**C**

**
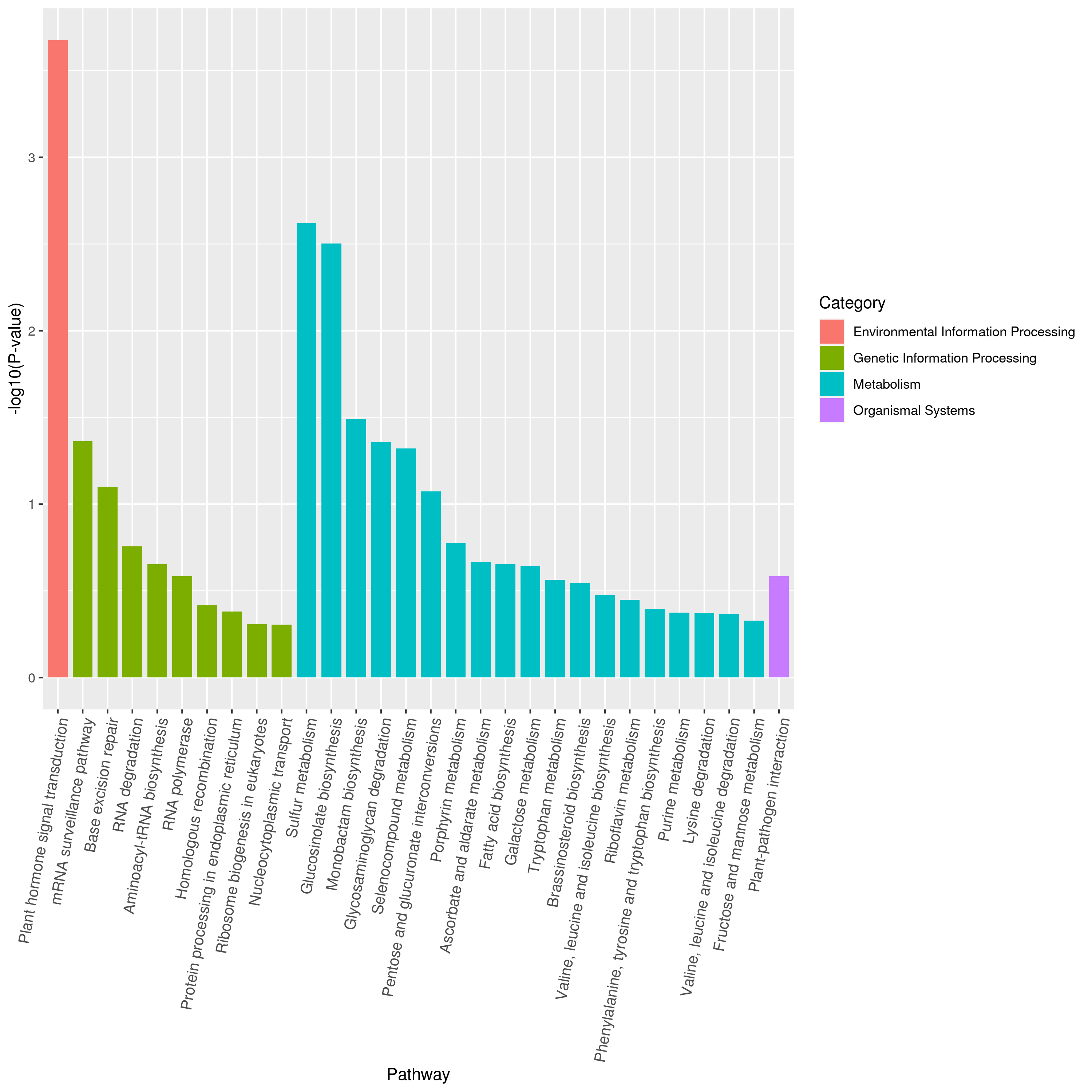
**

**D**

**
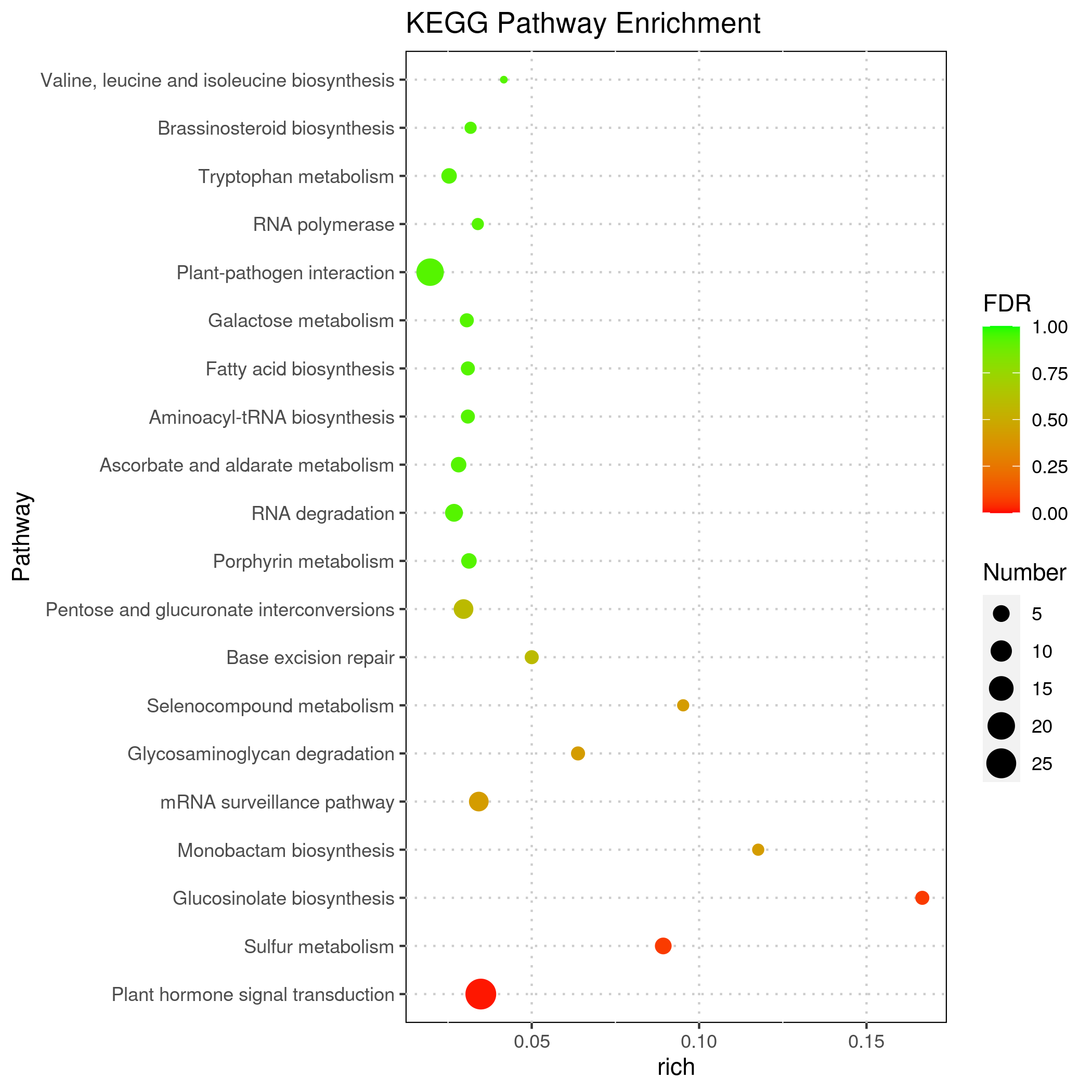
**

**Figure S1.** Gene Ontology (GO) and Kyoto Encyclopedia of Genes and Genomes (KEGG) pathway enrichment analyses of miRNAs identified in TLNVs. A：GO enrichment analysis presented as a bar chart, showing significantly enriched biological processes (BP), cellular components (CC), and molecular functions (MF) based on -log10(p-value). B：GO enrichment analysis visualized as a bubble plot, where bubble size indicates the number of genes and color indicates false discovery rate (FDR). C：KEGG pathway enrichment results shown as a bar chart, with pathways categorized by functional group. D： KEGG pathway enrichment results shown as a bubble plot, with bubble size representing gene count and color representing FDR.

|  |
| --- |
|  |
|  |
|  |
|  |
| 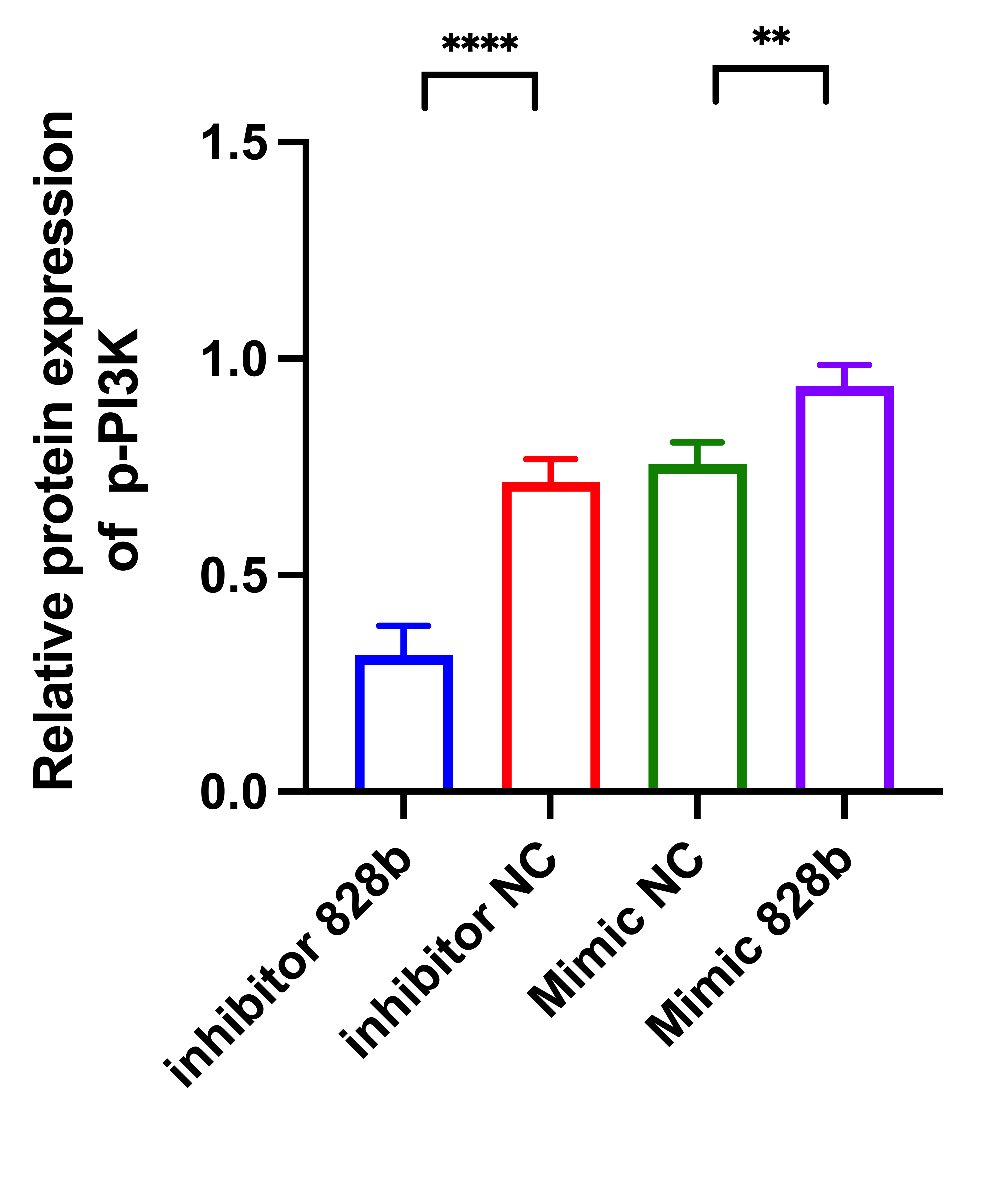  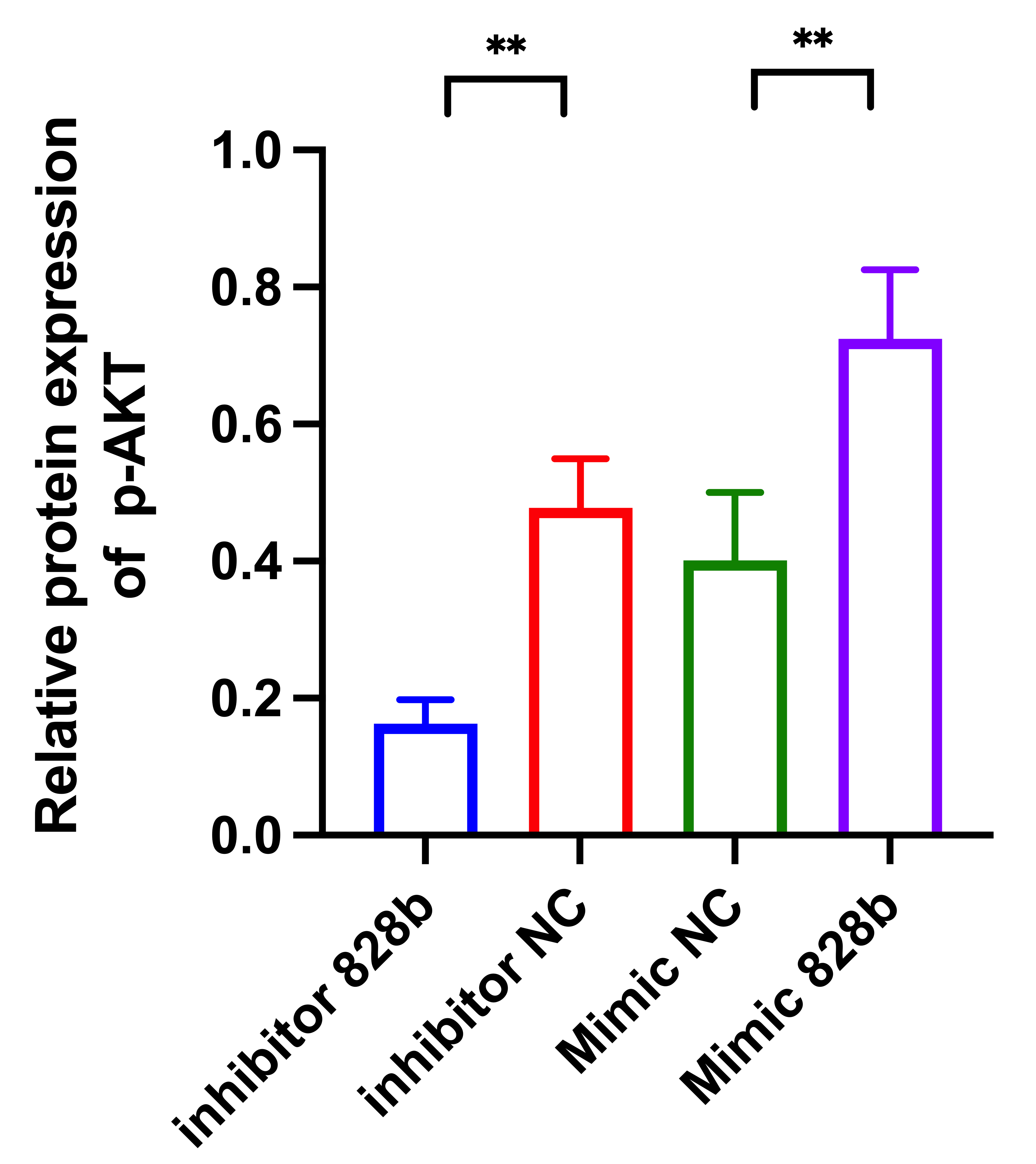  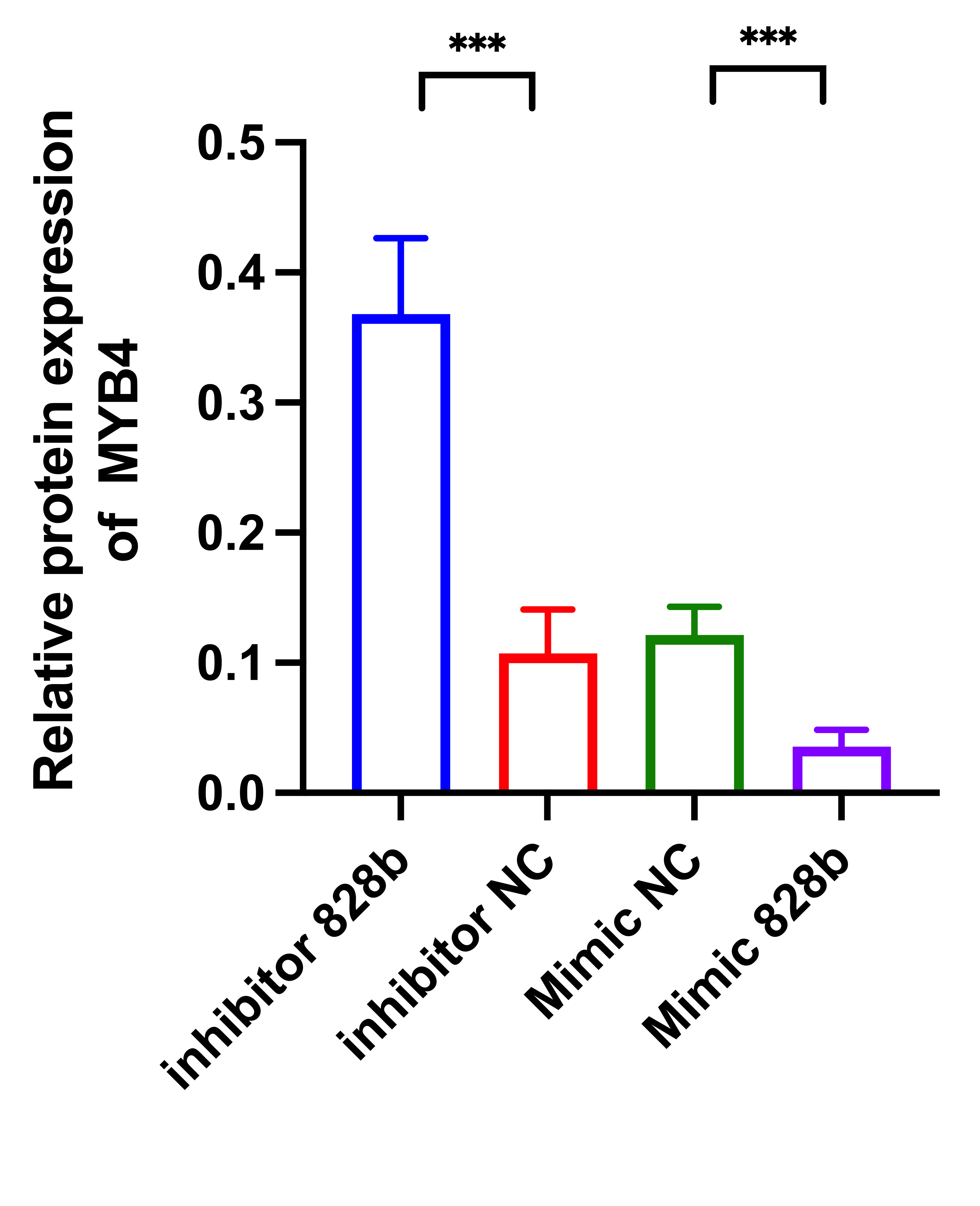 |

**Figure S2.** The effects of four treatments on the protein expression levels of p-PI3K, p-AKT and MYB4 in B16-F10 cells.


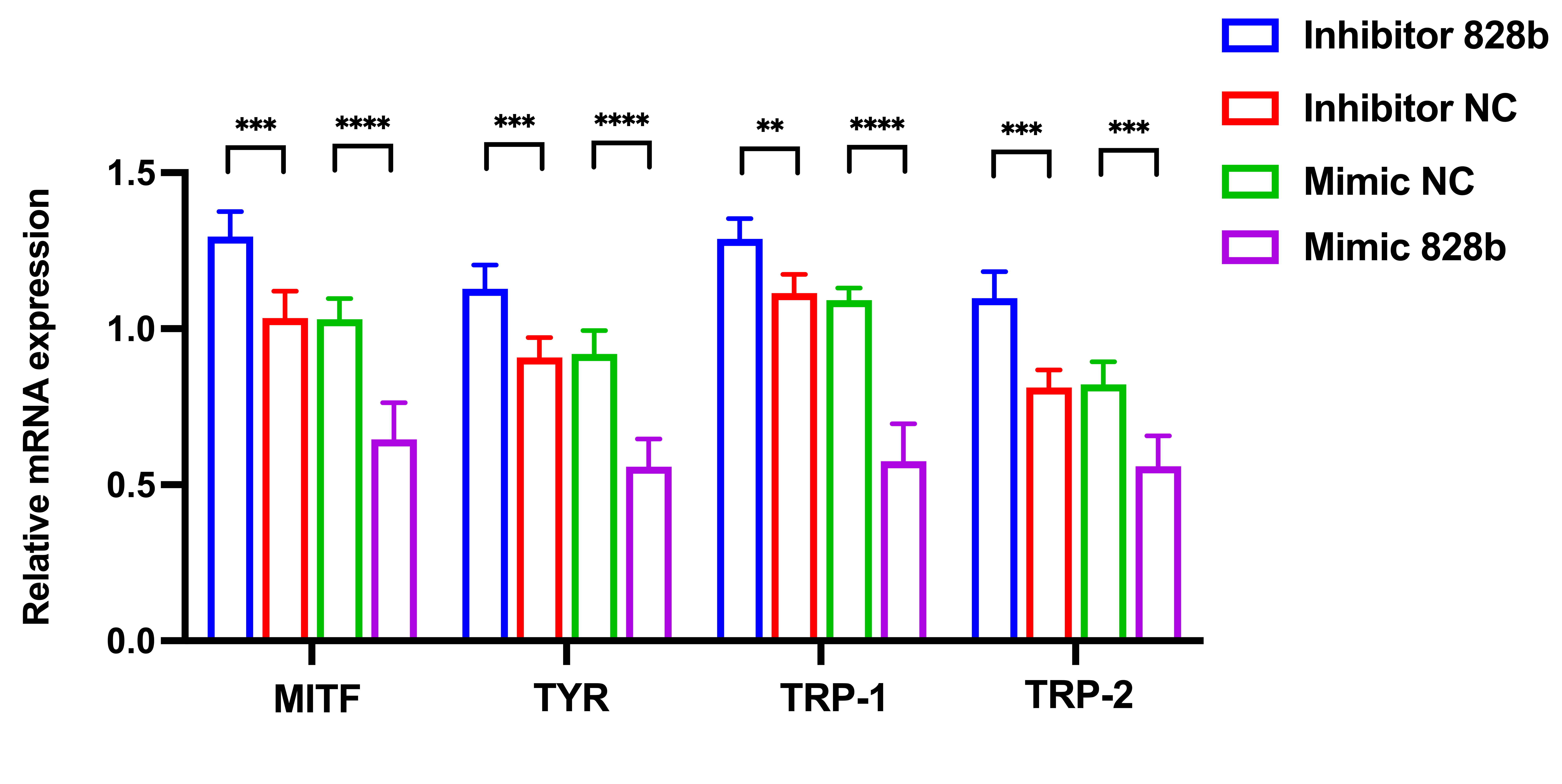


**Figure S3.** The effects of four treatments on the expression levels of MITF, TYR, TYP-1 and TRP-2 mRNA in B16-F10 cells.


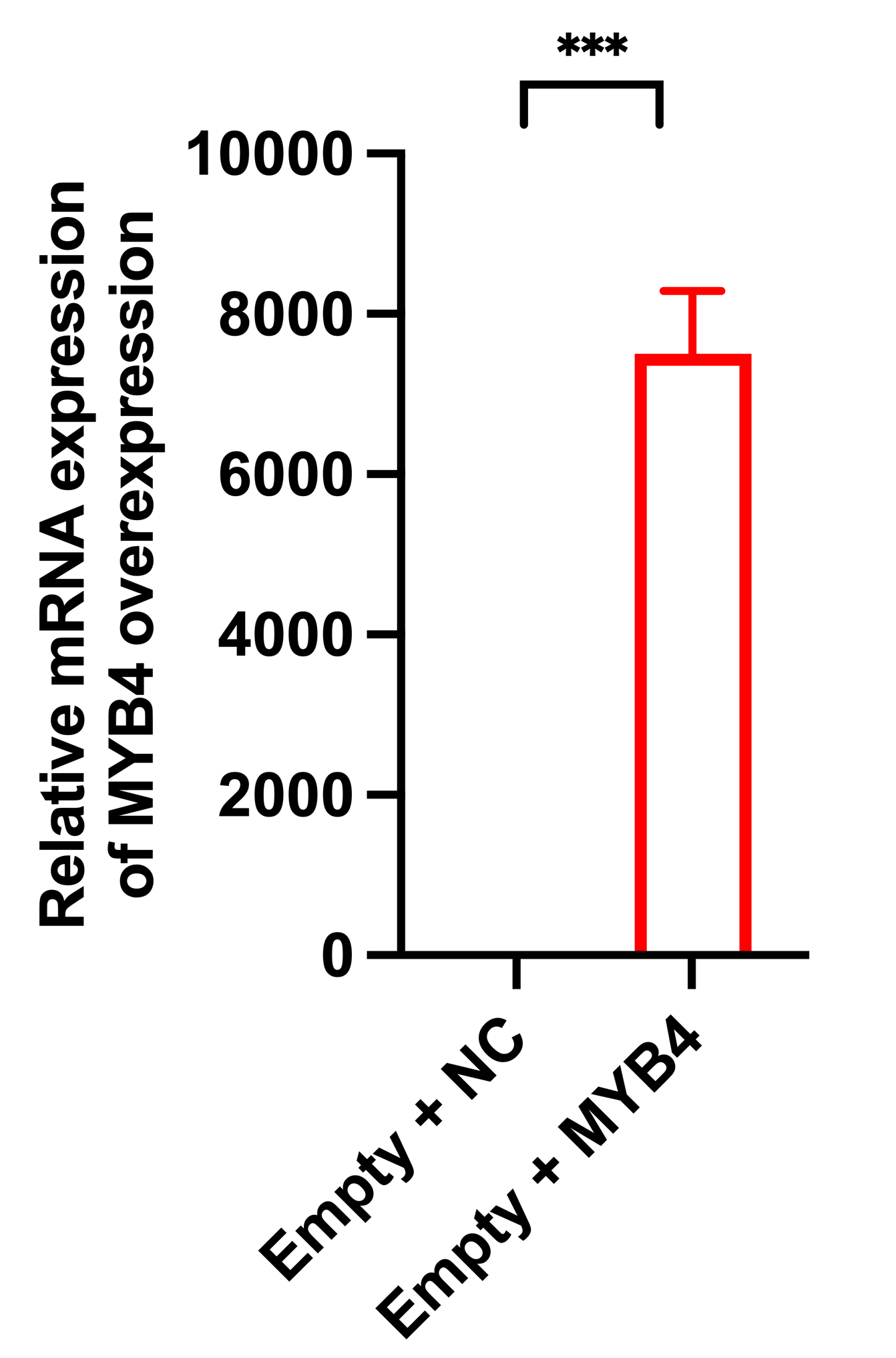


**Figure S4.** The overexpression level of MYB4 in B16-F10 cells.
